# ﻿Review of the millipede genus *Xystodesmus* (Diplopoda, Polydesmida), with seven new species from the southwestern part of Japan

**DOI:** 10.3897/zookeys.1231.141443

**Published:** 2025-03-12

**Authors:** Zoltán Korsós, Yasuyuki Nakamura

**Affiliations:** 1 Department of Zoology, Hungarian Natural History Museum, Baross u. 13, H-1088 Budapest, Hungary Hungarian Natural History Museum Budapest Hungary; 2 Department of Zoology, University of Veterinary Medicine Budapest, Rottenbiller u. 50, H-1077 Budapest, Hungary University of Veterinary Medicine Budapest Budapest Hungary; 3 Tropical Biosphere Research Center, University of the Ryukyus, Senbaru 1, Nishihara, Okinawa 903-0213, Japan The Museum of the University of the Ryukyus Nishihara Japan; 4 Fujukan, The Museum of the University of the Ryukyus, Senbaru 1, Nishihara, Okinawa 903-0129, Japan University of the Ryukyus Nishihara Japan

**Keywords:** Island geography, millipede colouration, new combinations, new species, Ryukyu Archipelago, Xystodesmidae

## Abstract

The genus *Xystodesmus* Cook, 1895 (most closely related to *Riukiaria* Attems, 1938) is retained as a taxon of xystodesmine millipedes with a relatively small body length (25–35 mm), male gonopods more complicated than just a forceps-like conformation, and with live colouration of grey-brown tergites with red, orange, or yellow paranotal spots. Seven new species of the genus (*Xystodesmusfasciatus***sp. nov.**, *X.keramae***sp. nov.**, *X.kumamotoensis***sp. nov.**, *X.kumeensis***sp. nov.**, *X.parvus***sp. nov.**, *X.rebekae***sp. nov.**, *X.sesokoensis***sp. nov.**) are described from the islands of Kyushu, Okinawa-jima, Kume-jima, Okinoerabu-jima, Aka-jima, Amami-O-shima, and Sesoko-jima, southwestern Japan. *Koreoaria* Verhoeff, 1937, **syn. nov.** is synonymised with *Xystodesmus* Cook, 1895, so *X.pallidus* (Verhoeff, 1937), **comb. nov.** (ex *Koreoariapallida*), and *X.amoenus* (Takakuwa, 1942), **comb. nov.** (ex *K.amoena*) are established. Furthermore, *X.variatus* (Pocock, 1895), **comb. nov.** (ex *Fontariavariata*), and *X.saltuosus* (Haga, 1968), **comb. nov.** (ex *Rhysodesmussaltuosus*) are re-evaluated and redescribed, based on re-examination of types and of freshly collected material (*X.variatus* only). All new species, as well as *X.martensii* (Peters, 1864), *X.nikkoensis* (Chamberlin & Wang, 1953), and *X.variatus* are illustrated with colour habitus photographs taken of live specimens, to facilitate field identification.

## ﻿Introduction

The millipede genus *Xystodesmus* Cook, 1895 belongs in the tribe Xystodesmini, subfamily Xystodesminae, family Xystodesmidae (Marek et al. 2014; Means et al. 2021b). As it was defined by Tanabe and Shinohara (1996) who had considered Harpaphini and Xystodesmini together, the tribe Xystodesmini – in addition to the four North American genera (*Harpaphe* Cook, 1904, *Isaphe* Cook, 1904, *Tubaphe* Causey, 1954, and *Thrinaphe* Shelley, 1993) – also consists of four more Far Eastern genera: *Levizonus* Attems, 1898, *Koreoaria* Verhoeff, 1937, *Riukiaria* Attems, 1938, and *Yaetakaria* Hoffman, 1949. The Xystodesmini also includes species formerly included in the North American tribes Chonaphini and Orophini based on the molecular phylogenetics of Means et al. (2021b). Of the four Far Eastern genera, *Levizonus* was previously revised by Tanabe (1994), and restricted only to two species: *L.montanus* (Takakuwa, 1941) and *L.takakuwai* (Verhoeff, 1941). *Koreoaria* is herewith synonymised with *Xystodesmus*, based on the examination of its type species *K.pallida* Verhoeff, 1937. The genus *Yaetakaria* is likely best considered as a synonym of *Riukiaria* (Shinohara 1977; Means et al. 2021b).

Tanabe and Shinohara (1996), when revising *Xystodesmus*, concluded that its closest relative is *Riukiaria*, and attempted to identify the synapomorphic characters of the two genera. This evolutionary sister-group relationship of *Riukiaria* and *Xystodesmus* was supported by the analysis by Means et al. (2021b). Tanabe and Shinohara (1996) recognised six species in *Xystodesmus*: *X.martensii* (Peters, 1864), *X.shirozui* (Takakuwa, 1942), *X.gracilipes* (Takakuwa, 1943), *X.serrulatus* (Miyosi, 1952), *X.nikkoensis* (Chamberlin & Wang, 1953), and *X.tokaiensis* Tanabe & Shinohara, 1996. However, during their analysis of several other populations, and also considering literature data, at the end they listed a number of “uncategorised populations” and possible synonyms, hence hypothesising several undescribed species of the genus. Masuda (2001) described *Xystodesmusyamamiensis* Masuda, 2001 from Honshu, Chita Peninsula, Aichi Prefecture, Japan. The description is not fully detailed, but the gonopod sketches clearly show a valid species of the genus.

In this paper we provide descriptions of seven new *Xystodesmus* species, two from Kyushu (Kagoshima, Kumamoto, and Oita prefectures) and five from the Central Ryukyu Archipelago (Kagoshima and Okinawa prefectures). The latter records considerably extend the distribution range of the genus to the south. In addition, we also provide redescriptions of four old and poorly known species: *Fontariavariata* Pocock, 1895, *Koreoariapallida* Verhoeff, 1937, *K.amoena* Takakuwa, 1942, and *Rhysodesmussaltuosus* Haga, 1968. With the re-examination of their types (except *K.amoena*, which is considered lost), and in the case of *F.variata* also with freshly collected material at hand, we assign them to *Xystodesmus*, thus increasing the known number of species in the genus to 18. The relationship between *Xystodesmus* and *Riukiaria* is also briefly reassessed.

## ﻿Materials and methods

Samples were collected by the authors on various occasions, in the framework of a comprehensive survey to investigate the millipede fauna of the Ryukyu Archipelago in the southwestern part of Japan. Denomination and writing style of the Japanese island names throughout the present paper follow Nakamura and Korsós (2010).

Type specimens of *Fontariavariata*, *Koreoariapallida*, *Rhysodesmussaltuosus*, and *Xystodesmusyamamiensis* were borrowed from the Natural History Museum, London (**NHMUK**), the Bavarian State Collection of Zoology (Zoologische Staatssammlung), München (**ZSMC**), and the National Museum of Nature and Science, Tokyo (**NSMT**).

Specimens were observed and drawn by the first author (ZK) with a Leica M125 stereomicroscope at the University of the Ryukyus. Terminology of xystodesmid gonopods follows Tanabe and Shinohara (1996) and Shelley and Smith (2018), with additional advice by R. L. Hoffman, W. A. Shear, and R. M. Shelley (in litt.). Total length of specimens was measured with a string, and midbody width on the 10^th^ segment under the microscope with an eyepiece reticule. Live photographs of specimens were taken in the field with a Nikon D90 digital camera, fitted with 60 mm Micro Nikkor lens and R1C1 macroflash system.

Type material of new species and other specimens are deposited in the Department of Zoology, Division of Terrestrial Invertebrates, National Museum of Nature and Science, Tokyo (**NSMT**), in Fujukan, the Museum of the University of the Ryukyus, Okinawa (**RUMF**), in the Myriapoda Collection of the Hungarian Natural History Museum, Budapest (**HNHM**), in the Natural History Museum of Denmark, Copenhagen (**NHMD**), and in the Virginia Museum of Natural History, Martinsville, USA (**VMNH**).

### ﻿Abbreviations (institute acronyms follow Sierwald and Reft 2004)

**NHMUK** Natural History Museum, London, United Kingdom

**HNHM**Myriapoda Collection of the Hungarian Natural History Museum, Budapest, Hungary

**NHMD** (formerly ZMUC) – Natural History Museum of Denmark, Copenhagen, Denmark

**NCSM** North Carolina State Museum of Natural Sciences, Raleigh, USA

**NSMT** Department of Zoology, Division of Terrestrial Invertebrates, National Museum of Nature and Science, Tokyo, Japan

**RUMF** Fujukan, the Museum of the University of the Ryukyus, Okinawa, Japan

**VMNH** Virginia Museum of Natural History, Martinsville, USA

**ZSMC** Bavaria State Collection of Zoology, München, Germany

## ﻿Taxonomy

**Family Xystodesmidae Cook, 1895**

**Subfamily Xystodesminae Hoffman, 1978**

**Tribe Xystodesmini Hoffman, 1980**

### ﻿Genus Xystodesmus

Taxon classificationAnimaliaPolydesmidaXystodesmidae

Cook, 1895

BEBBA818-E33E-56FF-8182-1673AC5166E7

Takakuwaia Verhoeff, 1936 (with the type species *T.furculigera* Verhoeff, 1936): synonymised by Hoffman (1956)
Cyphonaria Verhoeff, 1936 (with the type species C.scabra Verhoeff, 1936): synonymised by Hoffman (1980)
Phrurodesmus Takakuwa, 1943 (with the type species P.gracilipes Takakuwa, 1943): synonymised by Tanabe and Shinohara (1996)
Nikkonus Chamberlin & Wang, 1953 (with the type species N.nikkoensis Chamberlin & Wang, 1953): synonymised by Tanabe and Shinohara (1996)
Koreoaria Verhoeff, 1937, syn. nov.

#### Type species by original designation.

Polydesmus (Fontaria) martensii Peters, 1864.

#### Diagnosis.

The genus *Xystodesmus*, as a member of the tribe Xystodesmini, is characterised by the followings: small body size (less than 40 mm), posteriolateral corners of paranota usually acute, extending posteriorly beyond medial metatergal margin, presence of paranotal spots (red, orange, or yellow), and gonopods composed of coxa with prefemur and acropodite fused into a simple telopodite. In East Asia the tribe Xystodesmini is represented by five genera: *Koreoaria* Verhoeff, 1937 (herewith synonymised with *Xystodesmus*), *Levizonus* Attems, 1938, *Riukiaria* Attems, 1938, *Xystodesmus*, and *Yaetakaria* Hoffman, 1949. From these, based on gonopods, the most similar genus to *Xystodesmus* is *Riukiaria*, which share a common male gonopodal plan, usually with two well-developed processes (Tanabe and Shinohara 1996; Korsós et al. 2011). *Xystodesmus*, however, has various additional gonopodal appendages, while those of *Riukiaria* have none or only a few (its gonopod is more-or-less forceps-like). We also consider that colouration of live specimens is distinctive, with respect to their bright paranotal spots. All xystodesmids are strongly fluorescent when illuminated with ultraviolet light. Although traditional taxonomy is primarily based on gonopod morphology (Marek et al. 2014), the separation of the two genera is also supported by molecular phylogeny (Means et al. 2021b).

*Koreoaria* was introduced by Verhoeff (1937) with the South Korean species *K.pallida* (Verhoeff, 1937). He compared the new genus to *Pachydesmus* Cook, 1895, based on the superficially similar gonopod conformation (Verhoeff 1937). *Pachydesmus*, however, proved to be a strictly North American genus (Hoffman 1958) in its own tribe Pachydesmini (Hoffman 1980), and *Koreoaria* was considered belonging to the East Asian members of Xystodesmini (Tanabe and Shinohara 1996; Korsós et al. 2011). A second species, *Koreoariaamoena* (Takakuwa, 1942) was described, also from South Korea, and both species were proposed as “species possibly belonging to *Xystodesmus*” (Tanabe and Shinohara 1996: 1487). Observing the overall similarity of the type material of the type species of *Koreoaria*, here we take the opportunity to formalise the synonymy under *Xystodesmus*.

#### Species and distribution of *Xystodesmus*

(in order of date of description):

*X.martensii* (Peters, 1864): Kanto and Chubu regions, Honshu, Japan (Peters 1864: 3 as *Polydesmusmartensii*; Cook 1895: 5; Hoffman 1956: 97–99, figs 1–4; Tanabe and Shinohara 1996: 1480–1482, figs 2–9, 10A–E, 11A–E, 12A, B, 14, 17A–F)

*X.variatus* (Pocock, 1895), comb. nov.: Okinawa-jima Isl., Okinawa Pref., Japan (Pocock 1895: 361, figs 15, 15a as *Fontariavariata*)

*X.pallidus* (Verhoeff, 1937), comb. nov.: South Korea (Verhoeff, 1937: 319–320, fig. 8 as *Koreoariapallida*)

*X.shirozui* (Takakuwa, 1942): Tsushima and Iki Isl., also Danjo Isl. (Oshima, Meshima), Nagasaki Pref., Japan (Takakuwa 1942a: 239, fig. 5 as *Rhysodesmusshirozui*; Takakuwa 1954: 63–64, fig. 65; Miyosi 1959: 80, fig. 55; Minato 1973: 127–128, fig. A; Tanabe and Shinohara 1996: 1485–1487, figs 11M, N, 13C, D, 16C, D, 17O, P)

*X.amoenus* (Takakuwa, 1942), comb. nov.: Daegu, South Korea (Takakuwa 1942b: 362–363, fig. 5 as *Koreoariaamoena*)

*X.gracilipes* (Takakuwa, 1943): Ehime Pref., Shikoku, Japan (Takakuwa 1943: 604–605, fig. 2 as *Phrurodesmusgracilipes*; Takakuwa 1954: 84–85, figs 93, 94; Murakami 1965: 165, figs 1, 2; Tanabe and Shinohara 1996: 1484–1485, figs 11L, 13B, 16B, 17K)

*X.serrulatus* (Miyosi, 1952): Central part of Honshu and Shikoku (Tokushima Pref.), Japan (Miyosi 1952: 281, fig. 1 as *Rhysodesmusserrulatus*; Tanabe and Shinohara 1996: 1483–1484, figs 10G, 11H, I, 12D, 15D–I, 17I, J)

*X.nikkoensis* (Chamberlin & Wang, 1953): Yaku-shima Isl., Kagoshima Pref., Japan (the two type specimens from Honshu: Tochigi Pref., Nikko, are probably mislabeled) (Chamberlin and Wang 1953: 9, fig. 3 as *Nikkonusnikkoensis*; Tanabe and Shinohara 1996: 1484, figs 11J, K, 13A, 16A, 17L, M)

*X.saltuosus* (Haga, 1968), comb. nov.: Fukuoka Pref., Kyushu, Japan (Haga 1968 8, fig. 6a–e as *Rhysodesmussaltuosus*)

*X.tokaiensis* Tanabe & Shinohara, 1996: Shizuoka Pref., Honshu, Japan (Tanabe and Shinohara 1996: 1482–1483, figs 1, 10F, 11F, G, 12C, 15A–C, 17G, H)

*X.yamamiensis* Masuda, 2001: Aichi Pref., Honshu, Japan (Masuda 2001: 635–636, fig. 1A–H)

*X.fasciatus* sp. nov.: Satsuma Peninsula, Kagoshima Pref., Kyushu, Japan

*X.keramae* sp. nov.: Aka-jima Isl. (possibly also Tokashiki-jima Isl.), Okinawa Pref., Japan

*X.kumamotoensis* sp. nov.: Kumamoto and Oita Pref., Kyushu, Japan

*X.kumeensis* sp. nov.: Kume-jima Isl., Okinawa Pref., Japan

*X.parvus* sp. nov.: Okinoerabu-jima Isl., Kagoshima Pref., Japan

*X.rebekae* sp. nov.: Okinawa-jima Isl., Okinawa Pref., Japan

*X.sesokoensis* sp. nov.: Sesoko-jima Isl., Okinawa Pref., Japan

From the species list above, we did not have the opportunity to examine specimens of four species, *X.shirozui*, *X.gracilipes*, *X.serrulatus*, and *X.tokaiensis*, all occurring on the main islands of Japan (Honshu, Shikoku, and Kyushu) and adjacent islands, because of the loss of type material and/or the lack of fresh specimens. *Xystodesmusmartensii* and *X.yamamiensis* are also distributed in Honshu, but since we had freshly collected material and the type specimens, respectively, we could compare them to the other species and include them in our descriptions. For the former Korean genus *Koreoaria*, type material of *K.pallida* was available for comparison and redescription, but unfortunately the types of *K.amoena* could not be found; the majority of Y. Takakuwa’s material, kept after his death in 1960 by Y. Miyosi, is believed to have been lost after Miyosi’s death in 1995 (Tanabe and Shinohara 1996; Chao and Chang 2008). All the other *Xystodesmus* species in our review, including the new ones, occur in the southwestern part of Japan (Kagoshima and Okinawa prefectures).

### ﻿ Xystodesmus fasciatus sp. nov.

Taxon classificationAnimaliaPolydesmidaXystodesmidae

464AF9A5-7BBF-55A3-905B-6EE981C1C320

https://zoobank.org/DAAF356A-CB47-4987-A534-9E3CAA3285FD

Figs 1A–F , 16A

#### Type material.

***Holotype***: • male, Japan, Kyushu, Kagoshima Pref., Satsuma Peninsula, Hioki City, Fukiage Town, Yokura, *Cryptomeriajaponica* plantation, 15 m a.s.l., 31°30'46.4"N,130°22'31.1"E, 14 October 2009, leg. Z. Korsós and Y. Nakamura (NSMT-My 534). ***Paratypes***: • 1 male, and 1 juv. male, same locality and date as holotype (HNHM diplo-04540). 2 males, same locality and data as holotype (RUMF-ZD-00952 and 00953).

#### Diagnosis.

Medium-sized *Xystodesmus* species showing the general colour pattern plus a dark dorsal transversely banded appearance. Metatergites are smooth, shine, in contrast to that of *X.martensii* where they are conspicuously tuberculate. In terms of gonopods, the most similar species is *X.martensii* which also has a strong coxal apophysis, but in *X.fasciatus* sp. nov. it is almost hook-like; prefemoral process flat and wide, curving backwards, in *X.martensii* it is slender and straight. Acropodite with three small teeth at tip, whereas in *X.martensii* it has a cup-shaped process. *X.yamamiensis* has also a strong coxal apophysis, but its acropodite is strongly bifurcated and its prefemoral process is slender and tapering.

#### Description

(based on the two adult male specimens). Length 32–33 mm, midbody width with paranota 5.8 mm, metatergal length 1.9 mm, collum width 4.9 mm, median collum length 3.1 mm. Body sides between segments 5–13 parallel. Head smooth, with 1+1 frontal setae, epicranial suture distinct. Antennae straight, slender, first article globose, articles 2–4 subequal in length, 5 and 6 increasingly longer, 7 small, with length equal to width. Proterga completely smooth, metaterga very weakly wrinkled, each with a transverse depression in the middle. No trace of tubercles or punctation on metaterga. Collum subtrapezoid-shape, convex, its length double of metatergum 2, lateral edges directed ventrad, anteriolateral margin with weak ridge. Posteriolateral edge of paranota 2 and 3 rounded, of 4 slightly pointed, from 5 onwards triangular shaped, with strong half-circle excavation on the posterior edge of each metaterga. Pore formula normal, pores on segments 5, 7, 9, 10, 12, 13, 15, 16, 17, and 18, in lateral position on slightly swollen paranota.

Segments 14–19 gradually tapering, posteriolateral projections becoming more pointed, with strong excavations along their mesal side. Epiproct protruding, in lateral view distinctly curved, with two pairs of setae on small side tubercles, and with two setae apically; paraprocts (anal valves) smooth, with a pair of setae on obvious median ridges; hypoproct semicircular.

Bases of midbody leg pairs well separated (by 1.6 mm), sterna smooth and wide, pro- and metasterna well separated. Prefemur with well-developed ventral spine, increasingly larger from midbody legs onwards, femur ~ 2× as long as prefemur, almost straight, postfemur short and incrassate, tibia slender, long, nearly as long as tarsus, claw on pregonopodal legs flattened and curved, leaf-like, becoming normal on other legs towards the end of body.

Colour of living specimens on dorsal side vividly banded as a result of alternating pale greyish prozona and dark chocolate-brown metazona (Fig. 16A). Collum pale greyish in the middle and bordered with dark brown. Dark brown median stripe on dorsum from 4^th^ segments onward. Paranota bright reddish orange. Clypeus light brownish, underside of head, antennae, legs, epiproct, and whole ventral side pale, almost whitish. Colour in alcohol quickly bleaches, only the dorsal dark banded pattern remains visible.

**Figure 1.**
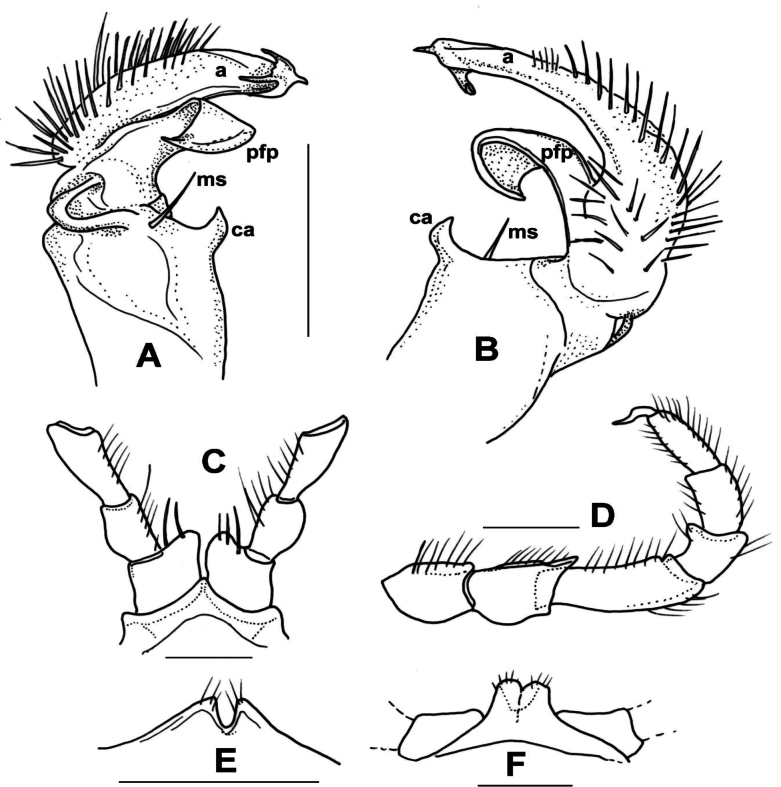
*Xystodesmusfasciatus* sp. nov., male paratype from Fukiage, Kagoshima Pref. **A, B** right gonopod, ventromesal and dorsolateral views, respectively **C** 2^nd^ legpair, anterior view **D** left 6^th^ leg, posterior view **E** sterna of 3^rd^ segment, anterior view **F** sterna of 4^th^ segment, anterior view. Abbreviations: a = acropodite, ca = coxal apophysis, ms = macroseta, pfp = prefemoral process. Scale bars: 1 mm.

**Male sexual characters.** Sterna of segments 3 and 4 (Fig. 1E, F) with a pair of protruding processes, on segment 3 small, widely separated from each other, with long apical setae; on segment 4 nearly as high as width of coxae, closely packed to each other, with short setae. Second leg pair with low but definite, blunt coxal processes (Fig. 1C). Other legs and sterna without extra modifications. Gonopods (Fig. 1A, B): Basically composed of two conspicuous processes, one considered as prefemoral process (*pfp*), and one as acropodite (*a*). Coxa strong, stout, nearly as long as wide, with well-developed, hook-like apophysis (*ca*) on its anteriomesal shoulder, and a large single apophyseal seta (*ms*). Prefemur stout, bent dorsad, densely setose, prefemoral process broad and flat, like a lamella, abruptly tapering to its tip which is pointed and curved backwards, in the direction of the coxal apophysis. Acropodite setose up to 2/3 of its length, gradually tapering towards its tip which is bent mesad, and terminates in two pointed branches connected to each other by a transparent, thin plate. At approximately the separation point of the two tips on the lateral side a small, there is a pointed process like a spur. Prostatic groove runs along the dorsomesal edge of the acropodite.

Female unknown.

#### Remarks.

The specimens were found in planted and managed *Cryptomeriajaponica* forest, in the deeper layers of mulch, in association with another xystodesmine millipede, *Riukiariacornuta* (Haga, 1968).

#### Etymology.

To emphasise the transversely banded appearance (= *fasciatus*, in Latin). Adjective, masculine.

### ﻿ Xystodesmus keramae sp. nov.

Taxon classificationAnimaliaPolydesmidaXystodesmidae

3A0918D5-4BC0-5BF2-B5F8-4CC1291D31F6

https://zoobank.org/029BA6FC-846B-4834-B32C-7D0F47AEB664

Figs 2A–D , 16B

#### Type material.

***Holotype***: • male, Japan, Central Ryukyus, Okinawa Pref., Okinawa Group, Aka-jima Isl., Mt. Ootake, shrine, 120 m a.s.l., mixed forest, 26°12'08.6"N, 127°16'32.5"E, 24 September 2010, leg. Z. Korsós (NSMT-My 535). ***Paratypes***: •1 male, 2 females (NSMT-My 536), 1 male, 2 females (RUMF-ZD-00945), 1 male, 1 female (HNHM diplo-04541) same locality and date.

#### Diagnosis.

*Xystodesmuskeramae* sp. nov. is a medium-sized *Xystodesmus* with typical colour pattern similar to *X.parvus* sp. nov. but differs from it by the well-developed coxal apophysis and the long, backwardly curved prefemoral process. It is also different from *X.serrulatus* where acropodite and prefemoral process are subequal in length, entirely straight, and very slender.

#### Description.

Length 26–32 mm, midbody width with paraterga 5.5–6.5 mm, midbody metatergal length 1.2–1.6 mm, collum width 4.4–4.8 mm, median collum length 1.9–2.4 mm. Body sides between segments 7–15 subparallel.

Head smooth, with 1+1 frontal setae, epicranial suture distinct. First antennal article sub-globose, 2^nd^ slightly clavate, otherwise subequal in length to straight articles 3–6, article 7 small, as long as wide, slightly tapering to its tip.

Pro- and metaterga completely smooth, transverse depression in metaterga clearly noticeable. Collum smooth, in dorsal view almost semicircular, posterior edge straight, slightly wavy, no marginal ridges, lateral corners triangular, directed posterior. Anterio-lateral edges of all paranota rounded, posterio-lateral corners of segment 2–4 in obtuse angle, posterior margins laterally bent forward. Small triangular projection starts from metaterga 5, only weakly increasing from segment 6 onwards, sublateral excavations lacking or only very shallow. Lateral sides of paranota slightly arched, outline of segments clearly delimited. Pore formula normal, pores in lateral central position on narrow paranota.

Segments 16–19 gradually tapering, caudal corners becoming strong and blunt. Epiproct in dorsal view triangular, in lateral view slightly curved downward, with four large setae on each lateral side on tubercles, projection with 2+2 setae apically; paraprocts slightly wrinkled, with two pairs of setae, upper ones on strong margin, lower ones on sides; hypoproct semicircular with two setae on tubercles.

Bases of midbody leg pairs clearly separated (by 1.2–1.6 mm in males, 1.5–1.7 mm in females), sterna smooth and wide, pro- and metasterna also clearly separated. Coxa short, as long as wide; prefemur ~ 1.8× longer than coxa, on male postgonopodal legs and on all female legs with well-developed ventral spine; femur strongly incrassate, only ~ 1.5× longer than prefemur; postfemur short, sub-globose, tibia and tarsus subequal in length, ~ 2× as long as wide; claw normal on all legs.

Colour of living specimens (Fig. 16B) pale brown, collum with dark margins, segments 2–4 with dark brown posterior margin, from 5 onwards medial part of metaterga with dark brown posterior margin; epiproct lighter. Head pale brown, antennae, legs, all paranota, and whole ventral side pale brown. Paranotal spots faded, hardly visible.

**Figure 2.**
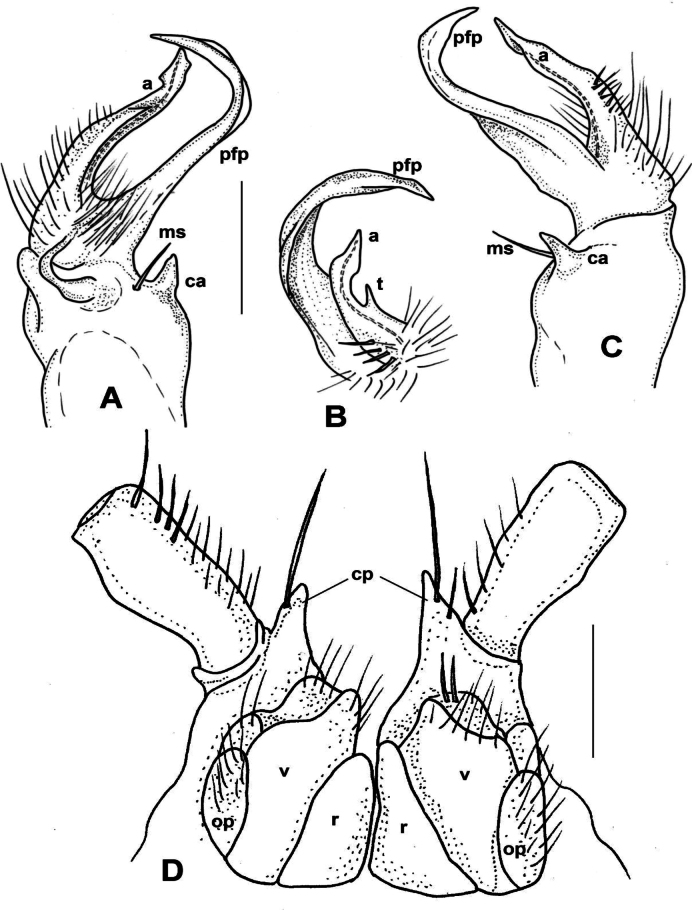
*Xystodesmuskeramae* sp. nov., Aka-jima Island, Okinawa Pref. **A–C** right gonopod of male paratype, mesal, lateral, and ventral (**B**, telopodite only) views, respectively **D** cyphopods with 2^nd^ leg pair of female paratype, posterior view. Abbreviations: a = acropodite, ca = coxal apophysis, cp = coxal projections, ms = macroseta, op = operculum, pfp = prefemoral process, r = receptacle, t = tooth, v = valve. Scale bars: 0.5 mm.

**Figure 3.**
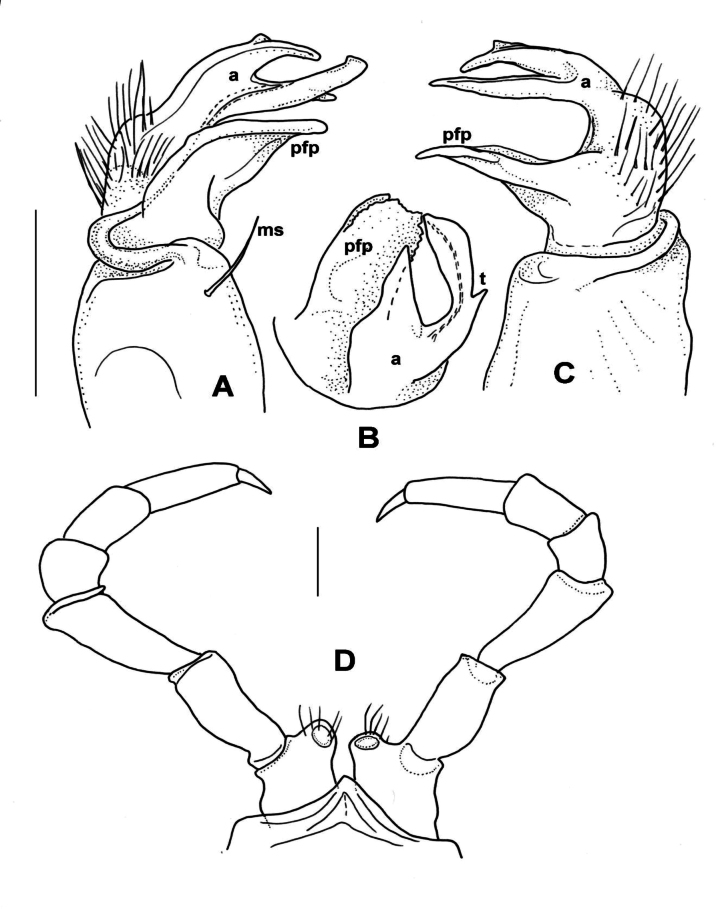
*Xystodesmuskumamotoensis* sp. nov., male paratype from Yatsushiro, Kumamoto Pref. **A–C** right gonopod, mesal, lateral, and acropodite (**B**) in ventral views, respectively. **D** 2^nd^ leg pair, posterior view. Abbreviations: a = acropodite, ms = macroseta, pfp = prefemoral process, t = tooth. Scale bars: 0.5 mm (**B**), 1 mm (**D**).

**Male sexual characters**. Second leg pair with strong, tubular coxal processes (as of *X.kumamotoensis* sp. nov., Fig. 3D) provided with numerous apical setae, sterna, and coxae of segments 4–6 and further legs without any modifications. Gonopods (Fig. 2A–C): Coxa stout, ~ 1.2× longer than wide, with strong but slender coxal apophysis, and a single macroseta (*ms*) next to it. Prefemur short, thick, densely setose on ventral side; prefemoral process (*pfp*) strong, longer than and parallel to acropodite, but its pointed tip bent ventrad and almost touching tip of acropodite; acropodite (*a*) short, more slender than prefemoral process, gradually tapering towards the somewhat broader, leaf-like tip, and with a small triangular tooth (*t*) on half way of its mesal side (Fig. 2B). Prostatic groove runs along the dorsomesal edge of acropodite.

**Female characters** (Fig. 2D). 2^nd^ leg pair with pointed coxal projections (*cp*); cyphopods behind closely packed in small aperture, very close to each other. Receptacula (*r*) on posterio-mesal side small, triangular, hardly setose; operculum (*op*) elongated with 3 several small setae; bursal valves (*v*) large, setose, with high mesal apices.

#### Remarks.

The type locality, Aka-jima Island is a member of the Kerama Islands, ~ 25 km west from the southern part of Okinawa-jima Island. Another member of the Kerama Islands is Tokashiki-jima Island, where a number of female *Xystodesmus* specimens were also found (see unidentified female at the end of the paper), probably the same as *X.keramae* sp. nov.

#### Etymology.

Named after the type locality which belongs in the Kerama Islands (a subgroup of the Okinawa Group), west of Okinawa-jima Isl., Okinawa Pref., Japan. Genitive, from *kerama*.

### ﻿ Xystodesmus kumamotoensis sp. nov.

Taxon classificationAnimaliaPolydesmidaXystodesmidae

75460D4A-08F5-5D2B-804B-FB2F72B52BE7

https://zoobank.org/5003BCBE-F05C-4FB2-BE92-F1FB906D1DD7

Figs 3A–D , 4A–C

#### Type material.

***Holotype***: • male, Japan, Kyushu, Kumamoto Pref., Yatsushiro City, Toyo Town, Kawamata, Kaerizaka, along prefectural road No. 25, 32°30'31.4"N, 130°45'42.5"E, 230 m a.s.l., 17 November 2008, leg. M. Nakano (NSMT-My 537). ***Paratypes***: • 1 male, 2 females, same locality and date (NSMT-My 538); • 1 male, Japan, Kyushu, Kumamoto Pref., Yatsushiro City, Toyo Town, Kawamata, Tsurukoba, along prefectural road No. 25, 32°28'45.9"N, 130°45'11.2"E, *Cryptomeriajaponica* forest, 700 m a.s.l., 18 September 2009, leg. T. Iihoshi (RUMF-ZD-00940); • 2 males, Japan, Kyushu, Oita Pref., Hita City, Kamitsue Town, Kawahara, 33°06'58.8"N, 130°57'50.9"E, along National Road No. 387, *Cryptomeriajaponica* forest, 14 November 2008, leg. T. Menda (HNHM diplo-04542, NHMD 1184732).

#### Diagnosis.

Relatively small *Xystodesmus* with colour pattern faded from preservation in ethanol but still typical for the genus. (Unfortunately, this is the only species of which we did not have live colour photo.) Gonopod completely lacks coxal apophysis as in the most similar species *X.shirozui* (Takakuwa, 1942), but differs from it by the widely bifurcated acropodite and the laminate, tongue-like prefemoral process.

#### Description.

Length 26–27 mm, midbody width with paraterga 4.8–5.0 mm, midbody metatergal length 1.2–1.3 mm, collum width 3.8–3.9 mm, median collum length 1.9 mm. Body sides between segments 5–16 subparallel.

Head smooth, with two frontal setae, epicranial suture distinct. Antennal articles straight, slender, first article short, 1.5× longer than wide, articles 2–6 subequal in length, 7 small, with length equal to width.

Proterga completely smooth, metaterga weakly wrinkled, each with a shallow transverse depression in the middle. Collum subtrapezoid-shape, arched, 1.5× longer than metatergum 2, lateral edges directed posterioventrad, anteriolateral margin with well visible ridge. Posterior edge of paranota 2–5 rounded, triangular projection only starts from segment 6 onwards, posterior margin of each metaterga straight, sublateral excavations hardly detectable. Lateral sides of paranota straight, almost giving a parallel straight outline to the body. Pore formula normal, pores in lateral position on narrow paranota.

Segments 17–19 gradually tapering, posteriolateral corners becoming more pointed. Epiproct protruding, in lateral view slightly curved, with two pairs of setae on small side tubercles, and with two setae apically; paraprocts smooth, with two pair of setae on obvious median ridges; hypoproct subtriangular.

Bases of midbody leg pairs clearly separated (by 1.1 mm), sterna smooth and wide, pro- and metasterna also clearly separated. Postgonopodal prefemora with well-developed ventral spine, increasingly larger from midbody legs onwards, femur 1.5× longer than prefemur, slightly bent proximad, postfemur short and curved, tibia slender, tarsus 1.5× longer than tibia, claws normal on all legs.

Colour of living specimens unknown. Preserved specimens show faded pattern typical for *Xystodesmus*: metaterga slightly darker, proterga paler, paranota with pale orangish/ yellowish spot. Clypeus light brownish, underside of head, antennae, legs, epiproct, and whole ventral side light pale white.

**Figure 4.**
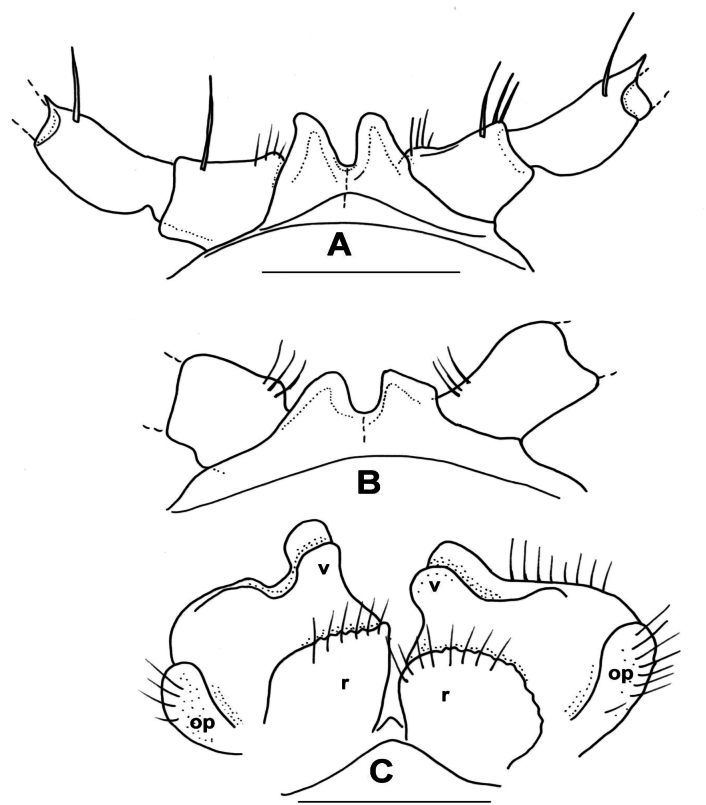
*Xystodesmuskumamotoensis* sp. nov. **A, B** male paratype from Yatsushiro, Kumamoto Pref. **A** sterna of 4^th^ segment, anterior view **B** sterna of 5^th^ segment, anterior view **C** cyphopods of female paratype from Yatsushiro, Kumamoto Pref., posterior view. Abbreviations: op = operculum, r = receptacle, v = valve. Scale bars: 1 mm (**A, B**); 0.5 mm (**C**).

**Male sexual characters**. Second leg pair with tubulous coxal processes (Fig. 3D), densely setose. Sterna of segments 4 and 5 (Fig. 4A, B) with a pair of protruding processes, on segment 4 longer, pointed, without setae, on segment 5 lower, widely separated from each other, without setae. Other legs and sterna without modifications. Gonopods (Fig. 3A–C): Coxa short and thick, almost as wide as long, no trace of apophyseal process, only a strong single seta (*ms*) on the anteriomesal side. Prefemur stout, nearly straight, densely setose, prefemoral process (*pfp*) broad and flat, lamellate, ~ 2× as song as wide, subrectangular from ventral view, distal margin slightly serrated (Fig. 3B). Acropodite (*a*) delimitated from prefemur by a weak constriction, its length only a little longer than prefemoral process, and soon divided into two branches subequal in length, directed anteriorly and gradually tapering to their pointed tips. Prostatic groove runs to the tip of the branch situated laterad, which is also provided with a small tooth (Fig. 3B, *t*) on it lateral side, best visible in situ from direct ventral view.

**Female sexual characters**. Cyphopods (Fig. 4C) situated in deep, joint excavation behind leg pair 2, loosely encapsulated in vulval sacs, well separated from each other. Receptacles (*r*) both on anterior and posterior side, densely setose; operculum (*op*) situated laterally, small, rounded, with numerous short setae; valves (*v*) hairless, mesally with conspicuous pointed apices, closely parallel to each other.

#### Remarks.

In the type locality and Toyo Town, 700 m a.s.l., *Riukiariasemicircularissemicircularis* (Takakuwa, 1941) and *R.cornuta* (Haga, 1968) were also found. In Hita City, *R.semicircularissemicircularis* and *Parafontariatonominea* (Attems, 1899) were also found together with the new species.

#### Etymology.

Named after the locality, Kumamoto Pref., Kyushu, Japan. Adjective, masculine.

### ﻿ Xystodesmus kumeensis sp. nov.

Taxon classificationAnimaliaPolydesmidaXystodesmidae

65DD3C9D-2079-502B-B2E4-79E5402F91B5

https://zoobank.org/2E374500-FA16-4E0D-85A8-56694CE56BEC

Figs 5A–F , 16C

#### Type material.

***Holotype***: • male, Japan, Central Ryukyus, Okinawa Pref., Okinawa Group, Kume-jima Isl., Kanegusuku–Self Defense Force base, 2.2 km from pref. road, 90 m a.s.l., 23 February 1992, leg. T. Tanabe (NSMT-My 539). ***Paratypes***: • 4 females (NSMT-My 540) same locality and date; • 1 male, 1 female, Japan, Central Ryukyus, Okinawa Pref., Okinawa Group, Kume-jima Isl., Uegusuku shrine, 26°22'35"N, 126°46'20"E, 235 m a.s.l., from roots of *Ficus* tree, 7 November 2011, leg. A. and Z. Korsós (RUMF-ZD-00946 [male] and -00947 [female]); • 2 males, Japan, Central Ryukyus, Okinawa Pref., Okinawa Group, Kume-jima Isl., Daruma-yama forest road, 26°22'07"N, 126°45'50"E, 204 m a.s.l., 9 November 2011, leg. A. and Z. Korsós (HNHM diplo-04543).

#### Other non-type material.

18 juveniles, same locality and date as holotype (NSMT-My 541).

#### Diagnosis.

Medium-sized *Xystodesmus*, male with essentially simple, two-branched gonopod, and a small, isolated coxal apophysis. Most similar to *X.rebekae* sp. nov., *X.gracilipes*, and *X.nikkoensis*, but differs from them by the presence of a coxal apophysis and by the configuration of the prefemoral and tibiotarsal processes. Prefemoral process is strongly twisted, longer than acropodite, at approximately midpoint with a small tooth, whereas in all the three others it is straight, slender, and subequal to acropodite, without tooth. In *X.nikkoensis*, acropodite has ventrally a small triangular process which is missing in *X.kumeensis* sp. nov.

#### Description.

Length 30–32 mm, midbody width with paraterga 5.5–6.2 mm, midbody metatergal length 1.4–1.8 mm, collum width 4.2–4.9 mm, median collum length 1.9–2.4 mm. Body sides between segments 5–14 subparallel.

Head smooth, epicranial suture distinct. Antennal articles slightly clavate, first article sub-globose, articles 2–6 subequal in length, 2 and 3 are most clavate, 4–6 almost straight, article 7 small, as long as wide, slightly tapering to its tip.

Pro- and metaterga completely smooth, transverse depression in metaterga hardly noticeable. Collum in dorsal view almost oval, edges rounded, with only very weak ridges, lateral corners directed ventrad, length along median line ~ 2× as long as metatergum 2. Anterior edge of paranota 2–4 rounded, posterior edge rounded, lacking projection. Posteriolateral triangular projection starts on metaterga 5, from 6 onwards increasingly pointed, sublateral excavations on caudal margin of midbody segments strong, semicircular. Lateral sides of paranota arched, outline of segments clearly delimited. Pore formula normal, pores in lateral central position on narrow paranota.

Segments 15–19 gradually tapering, caudal corners becoming more pointed. Epiproct protruding, in lateral view slightly curved, thick, with 2+2 large setae on each lateral side on tubercles, projection with 2+2 setae apically (i.e., 6 pairs of setae altogether); paraprocts smooth, with two pair of setae on strong median ridges; hypoproct semicircular with two setae on small tubercles.

Bases of midbody leg pairs well separated (by 1.2 mm in male, 1.6–1.8 mm in females), sterna smooth and wide, pro- and metasterna well separated. Coxa short, just a little bit longer than wide; prefemur ~ 1.2× longer, on postgonopodal legs with well-developed ventral spine; femur 2× as long as coxa, incrassate; postfemur short again like coxa, tibia, and tarsus subequal in length, both ~ ½ as long as femur; claws normal on all legs.

Colour of living specimens pale brown, almost yellowish, paranotal spots hardly visible (Fig. 16C). Preserved specimens show faded pattern typical for *Xystodesmus*: metaterga slightly darker, proterga paler, clypeus light brownish, underside of head, antennae, legs, epiproct, and whole ventral side yellowish white.

**Figure 5.**
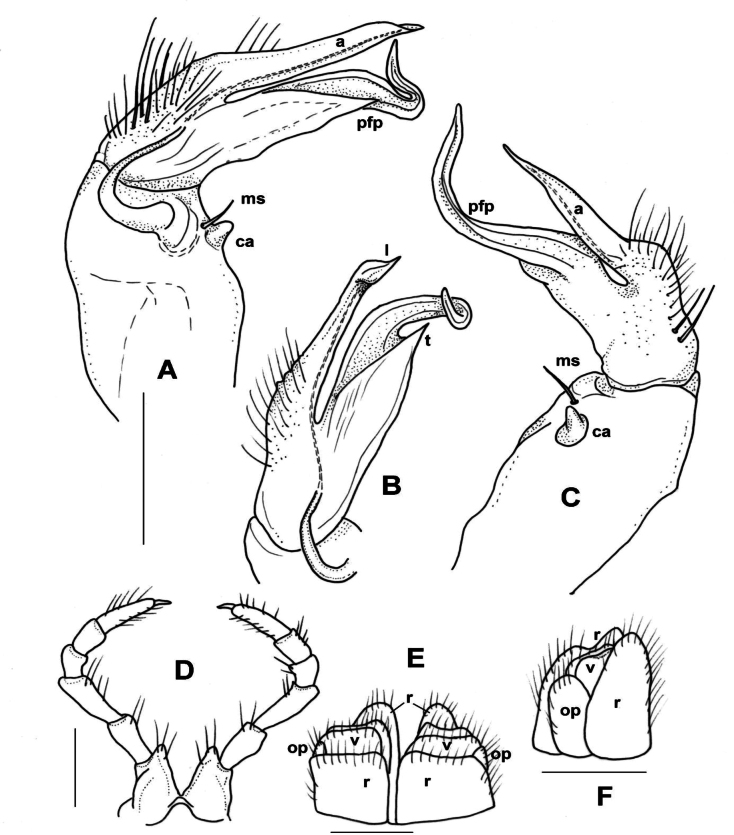
*Xystodesmuskumeensis* sp. nov., paratypes from Kume-jima Island, Okinawa Pref. **A–C** right gonopod of male paratype, mesal, lateral, and ventromesal (**B**, telopodite only) views, respectively **D** 2^nd^ legpair of female paratype, anterior view **E** cyphopods, posterior view **F** left cyphopod, anteriolateral view. Abbreviations: a = acropodite, ca = coxal apophysis, l = lamella, ms = macroseta, op = operculum, pfp = prefemoral process, r = receptacle, t = tooth, v = valve. Scale bars: 0.5 mm (**A–C, F**); 1 mm (**D**); 0.4 mm (**E**).

**Male sexual characters**. Second leg pair with tubulous coxal processes, sterna of segments 4–6 and further legs without any modifications. Gonopods (Fig. 5A–C): Coxa stout, ~ 1.5× longer than wide, coxal apophysis (*ca*) present in form of a small bump emerging from a circular field, next to a short, thick apophyseal macroseta (*ms*). Prefemur stout, subparallel-sided, densely setose only on ventral side; prefemoral process (*pfp*) slender, longer than acropodite, strongly curved into a sharp, pointed tip, at approximately midpoint with a widely based, triangular side tooth (Fig. 5B, *t*); acropodite (*a*) slim, more slender than prefemoral process, only ~ 2/3 of its length, almost straight, gradually tapering towards tip, slightly broadening before pointed tip into a leaf-like lamella (Fig. 5B, *l*). Prostatic groove runs straight on the mesal side of acropodite.

**Female sexual characters**. Coxae of 2^nd^ leg pair with a pair of small, pointed, setose projections (Fig. 5D). Cyphopods (Fig. 5E, F) situated in deep, joint aperture behind leg pair 2, loosely encapsulated in vulval sacs, well separated from each other. Receptacula (*r*) both on anterior and posterior side, densely setose; anterior receptaculum low, broad, posterior one high, slender, higher than bursal valves; operculum (*op*) small, rounded, situated laterad, in anterior view hardly visible; bursal valves (*v*) equal shaped, broad, rectangular, with short setae, without pointed apices.

#### Remarks.

The species was treated as an uncategorised population under *Xystodesmus* in Tanabe and Shinohara (1996: Cluster I, figs 7, 11Q, 16H, 17R). The individuals were found on Kume-jima Island in mixed *Pinusluchuensis* and broad-leaved evergreen forest.

#### Etymology.

Named after the collecting locality, Kume-jima Island, west of Okinawa-jima Isl., where the species is probably endemic to. Adjective, masculine.

### ﻿ Xystodesmus parvus sp. nov.

Taxon classificationAnimaliaPolydesmidaXystodesmidae

8760936E-FEE4-5BE0-B394-378280168056

https://zoobank.org/3BE0CB3D-3C8A-41B4-9C7F-871361BF3C2B

Figs 6A–E , 16D

#### Type material.

***Holotype***: • male, Japan, Central Ryukyus, Kagoshima Pref., Amami Group, Okinoerabu-jima Isl., Oyama botanical garden, 27°21'56.4"N, 128°34'00.0"E, 240 m a.s.l., 13 June 2010, leg. Z. Korsós and Y. Nakamura (NSMT-My 542). ***Paratypes***: • 4 females (NSMT-My 543), 1 male (RUMF-ZD-00941), 3 females (RUMF-ZD-00942), same locality and date as holotype.

#### Diagnosis.

Differs from its congeners by the small body size (length under 23 mm), and by the male gonopods lacking coxal apophysis and having two simple branches (prefemoral process and acropodite) subequal in length and bent parallel together. The closest species is the similarly small *X.sesokoensis* sp. nov., but its prefemoral process has two small branches at tip, and its acropodite is longer, the end is thicker with a small tooth.

#### Description.

Length 19–22 mm, midbody width with paraterga 4.1–4.2 mm, midbody metatergal length 0.9–1.1 mm, collum width 3.1–3.2 mm, median collum length 1.3–1.4 mm. Body sides between segments 5–15 subparallel.

Head smooth, with two frontal setae, epicranial suture distinct. Antennal articles slightly clavate, first article sub-globose, articles 2–6 subequal in length, article 7 small, as long as wide.

Collum in dorsal view oval, edges rounded, with well-developed anterior ridge, laterally even stronger like paranotum, corners rounded. Pro- and metaterga completely smooth, transverse depression in metaterga short but noticeable. Anterio-lateral edge of paranota rounded, posterio-lateral corner of segments 2 and 3 rounded, lacking projection; on 4 with small projection, from 5 increasingly pointed, triangular, sublateral excavations on posterior margin becoming stronger. Lateral sides of paranota straight, outline of segments clearly delimited. Pore formula normal, pores in lateral central position on narrow paranota.

Segments 16–19 gradually tapering, posterior corners becoming obtuse, sublateral excavations disappearing. Epiproct in dorsal view triangular, in lateral view slightly curved ventrad, with 4+4 large setae on tubercles on lateral sides, projection with 2+2 apical setae; paraprocts smooth, with two pairs of setae, upper ones on margin, lower ones on side; hypoproct semicircular with two setae on small tubercles.

Bases of midbody leg pairs well separated (by 1.2 mm in male, 1.6–1.8 mm in females), sterna smooth and wide, pro- and metasterna fused. Coxa short, rectangular; prefemur ~ 2× as long as wide, with well-developed ventral spine; femur 1.5× longer than prefemur, incrassate; postfemur small, sub-globose, tibia slender, approximately same length as postfemur, tarsus 2× as long as tibia, slender, tapering towards small, curved claw.

Colour of living specimens (Fig. 16D) pale brown, collum, segments 2, 18, 19, and epiproct darker, all metaterga with dark posterior margin. Underside of head, antennae, legs, and whole ventral side pale white. Sides of collum and all paranota (except segment 19) with faint, pinkish-orangish spots.

**Figure 6.**
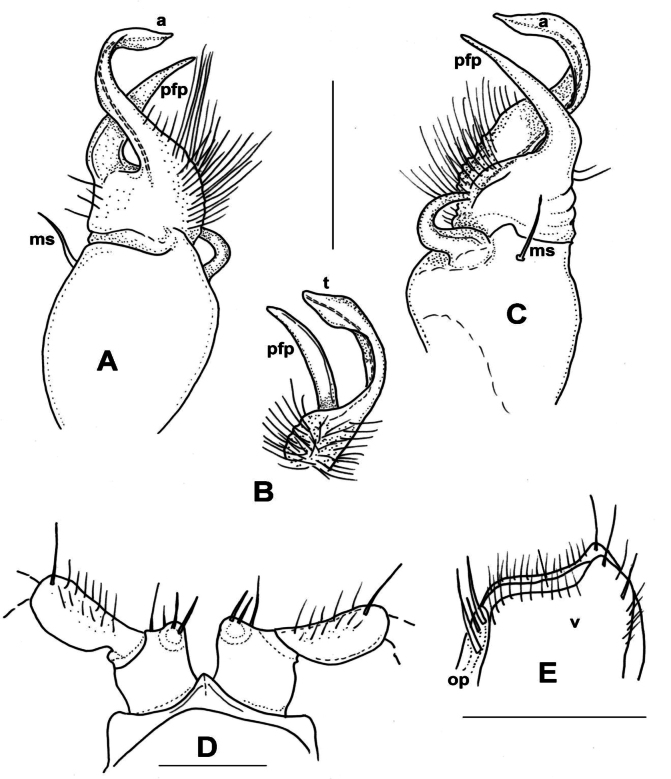
*Xystodesmusparvus* sp. nov., paratypes from Okinoerabu-jima Island, Kagoshima Pref. **A–C** right gonopod of male paratype, lateral, mesal, and ventral (**B**, telopodite only) views, respectively **D** 2^nd^ leg pair (from femur omitted) of male paratype, anterior view **E** left cyphopod of female paratype (receptaculum omitted), anterior view. Abbreviations: a = acropodite, ms = macroseta, op = operculum, pfp = prefemoral process, t = tip of acropodite, v = valve. Scale bars: 0.5 mm.

**Male sexual characters**. Second leg pair with small coxal processes provided with three or four strong setae (Fig. 6D), sterna of segments 4–6 and further legs without any modifications. Gonopods (Fig. 6A–C): Coxa stout, ~ 1.2× longer than wide, coxal apophysis completely lacking, apophyseal macroseta (*ms*) relatively small. Prefemur short, stout, parallel-sided, densely setose on ventral side, with a few setae on dorsal side; prefemoral process (*pfp*) slender, shorter than acropodite, bent ventrad and ending in a long, pointed tip; acropodite (*a*) slightly broader and longer, bending subparallel to prefemoral process, gradually tapering towards somewhat broader, leaf-like tip (*t*) (Fig. 6B). Prostatic groove runs along mesal side of acropodite.

**Female sexual characters**. Cyphopods (Fig. 6E) situated in deep, joint aperture behind leg pair 2, loosely encapsulated in vulval sacs, well separated from each other. Receptacula (not shown in figure) on both anterior and posterior side, setose only along margins, with pointed mesal tips; operculum (*op*) elongated with three strong setae; bursal valves (*v*) hidden between high receptacula, with short setae only.

#### Remarks.

The new species was found in a protected botanical garden on the top of the central hill on the 93 km^2^ Okinoerabu-jima Island. The vegetation cover is subtropical broad-leaved evergreen forest, and the specimens were collected in the deep, humid, top-soil litter layer. No sympatric species of millipede were found.

#### Etymology.

Named after its small size (*parvus*, in Latin). Adjective, masculine.

### ﻿ Xystodesmus rebekae sp. nov.

Taxon classificationAnimaliaPolydesmidaXystodesmidae

B4C9232A-ADFA-5D4B-8FC3-1C3D6C6948F5

https://zoobank.org/204CFBCA-2A67-4E13-A922-AE529E4A1E71

Figs 7A–D , 16E

#### Type material.

***Holotype***: • male, Japan, Central Ryukyus, Okinawa Pref., Okinawa Group, Okinawa-jima Isl., Motobu Peninsula, Mt. Yae-dake, 26°38'17.5"N, 127°55'37.9"E, 390 m a.s.l., 30 December 2009, leg. R. Korsós, Z. Korsós and Y. Nakamura (NSMT-My 544). ***Paratypes***: • 6 males and 2 females, same locality and date (1 male, 1 female NSMT-My 545; 1 male, 1 female RUMF-ZD-00943, RUMF-ZD-00944; • 1 male HNHM diplo-04544; • 1 male VMNH112447, 1 male NHMD 1184733); • 1 juv. male, same locality, 8 November 2010, leg. Z. Korsós and Y. Nakamura (No. 285) (HNHM diplo-04545).

#### Diagnosis.

Medium to relatively large *Xystodesmus* with typical colour pattern, dark brown terga and bright orange paranotal spots. With its simple, two-branched male gonopod completely lacking coxal apophysis and sternal modifications on pregonopodal legs *X.rebekae* sp. nov. differs from all its congeners. Most similar species are *X.variatus* and *X.gracilipes* which also have only two gonopodal processes, but *X.rebekae* sp. nov. has a longer, S-shaped femoral process and a shorter, slimmer acropodite.

#### Description.

Length 29–33 mm, midbody width with paraterga 6.6–7.3 mm, midbody metatergal length 1.5–1.8 mm, collum width 5.3–5.8 mm, median collum length 2.5–2.7 mm. Body sides between segments 5–14 subparallel.

Head smooth, with two frontal setae, epicranial suture distinct. Antennal articles more-or-less straight, slender, first article sub-globose, articles 2 and 3 subequal in length, 4–6 also subequal but slightly longer than 2 and 3, 7 small, little wider than long.

Pro- and metaterga completely smooth, transverse depression in metaterga hardly noticeable, but on midbody segments with two weak lateral swellings. Collum subtrapezoidal, arched, ≤ 2× longer than metatergum 2, lateral edges directed posterioventrad, anteriolateral margin with well visible ridge. Anterior edge of paranota 2–4 slightly rounded, almost right-angled, posterior edge rounded, without projection. Triangular projection behind on metaterga 5, from 6 onwards increasingly pointed, sublateral excavations on posterior margin of midbody segments already strong, semicircular. Lateral sides of paranota arched, outline of segments clearly delimited. Pore formula normal, pores in lateral central position on narrow paranota.

Segments 15–19 gradually tapering, posterior corners becoming more pointed. Epiproct protruding, in lateral view slightly curved, with two large setae on each lateral side on tubercles, projection with two pairs of setae on small side tubercles, two pairs in dorsolateral position, and 2+2 setae apically (8 pairs of setae altogether); paraprocts smooth, with two pair of setae on obvious median ridges; hypoproct subtrapezoidal with two setae on small tubercles.

Bases of midbody leg pairs well separated (by 1.3–1.5 mm), sterna smooth and wide, pro- and metasterna well separated. Coxa short, almost as wide as long; prefemur 1.2× longer, on postgonopodal legs with well-developed ventral spine; femur 1.2× longer than prefemur, slightly bent proximad; postfemur, tibia, and tarsus subequal in length, all ~ ½ as long as femur; claws normal on all legs.

Colour of living specimens (Fig. 16E) generally dark brown appearance with bright orange paranotal spots. Proterga slightly paler, metaterga with two circular lateral spots. Preserved specimens paler, but the circular markings and the yellowish paranotal spots still noticeable. Clypeus pale brownish, underside of head, antennae, epiproct, and whole ventral side pale whitish, legs pale brown.

**Figure 7.**
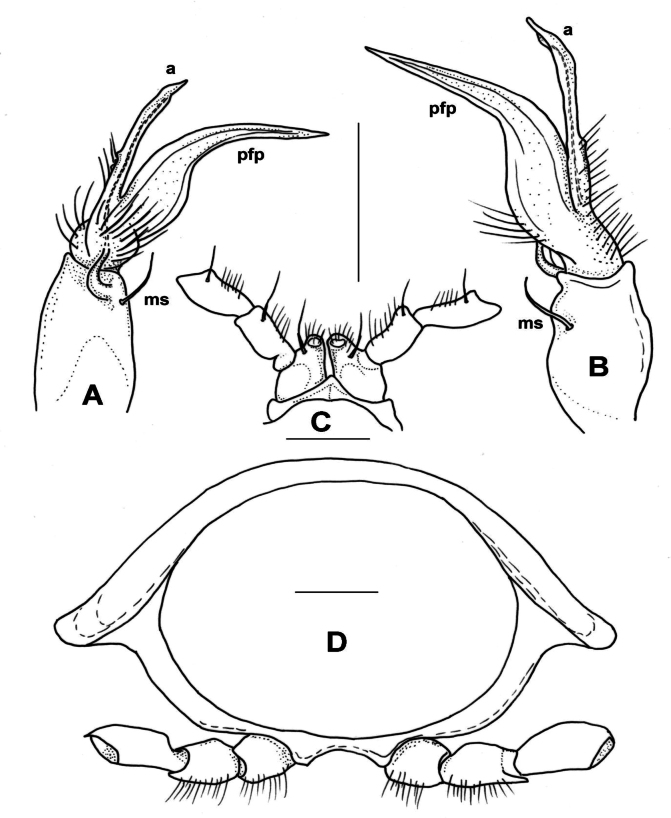
*Xystodesmusrebekae* sp. nov., male paratype from Mt. Yae-dake, Okinawa-jima Island, Okinawa Pref. **A, B** right gonopod, ventromesal and dorsolateral views, respectively **C** 2^nd^ legpair (from tibia omitted), anterior view **D** body cross section, 6^th^ segment, anterior view. Abbreviations: a = acropodite, ms = macroseta, pfp = prefemoral process. Scale bars: 1 mm.

**Male sexual characters**. Second leg pair (Fig. 7C) with small tubulous coxal processes, ~ 1/3 length of prefemora, sterna of segments 4–6 without any modifications (Fig. 7D). Gonopods (Fig. 7A, B): Coxa long and slender, ~ 2× as long as wide, coxal apophysis completely lacking, only a single apophyseal macroseta (*ms*) on anteriomesal side. Prefemur slender, bottle-shaped, densely setose; prefemoral process (*pfp*) long, longer than acropodite, drawn S-shaped, smooth, without spines or spurs, gradually tapering and ending in a pointed tip. Acropodite (*a*) slim, subparallel-sided, ~ 2/3 in length of prefemoral process, slightly spatulate before pointed tip where prostatic groove opens. In situ both branches (acropodite and prefemoral process) directed ventromesad, and those of the two gonopods crossing each other.

**Female sexual characters**. Cyphopods deeply embedded in joint aperture closely behind leg pair 2, encapsulated in separate vulval sacs. Receptacula on both anterior and posterior side, subtriangular, setose on entire surface; operculum oval, narrow; valves round, without projected tips, with short setae along margins.

#### Remarks.

The individuals were collected in broad-leaved forest ground, always under loose stones. In the same habitat, but in the litter, *Riukiariaholstii* (Pocock, 1895) and *Riukiarianeptuna* (Pocock, 1895) were also found. *X.rebekae* sp. nov. seems to be confined to the single locality which is a limestone hill with medium altitude (350–400 m) (relatively high for Okinawa-jima Isl.).

#### Etymology.

Named after Rebeka Korsós, daughter of ZK, who found the first specimen in the type locality. Genitive noun derived as a matronym.

### ﻿ Xystodesmus sesokoensis sp. nov.

Taxon classificationAnimaliaPolydesmidaXystodesmidae

DDE6D293-4743-517D-BE06-0AFBE56CDCCF

https://zoobank.org/4777B6CE-6F41-4F35-B67F-B2ABF3922444

Figs 8A–C , 16F

#### Type material.

***Holotype***: • male, Japan, Central Ryukyus, Okinawa Pref., Okinawa Group, Sesoko-jima Isl., Ominebaru, 26°38'36.7"N, 127°52'4.7"E, 11 March 2012, leg. Z. Korsós and K. Watanabe (NSMT-My 546). ***Paratypes***: • 1 male and 1 juvenile, same locality and date as holotype (HNHM diplo-04546).

#### Diagnosis.

A small-sized *Xystodesmus*, with typical colour pattern, pale brown with orange paranotal spots. In its adult size, the most similar species is *X.parvus* sp. nov. that has two simple branches whereas *X.sesokoensis* sp. nov. has a prefemoral process with two small branches at tip, and a long, bending acropodite with a thickened end and a small tooth. Gonopods are somewhat similar to those of *X.fasciatus* sp. nov. but are larger, with a strong coxal apophysis, and its prefemoral process is much wider.

#### Description.

Total body length 19–20 mm, midbody paratergal width 3.7 mm, length 0.8 mm, collum width 2.9–3.0 mm, length 1.3 mm. Body sides between segments 5–15 subparallel.

Head smooth, with two frontal setae, epicranial suture distinct. Antennal articles slightly clavate, first article sub-globose, articles 2–6 subequal in length, article 7 small, as long as wide.

Collum in dorsal view oval, edges rounded, with small anterior ridge, corners rounded. Pro- and metaterga completely smooth, transverse depression not visible. Segments 2 and 3 rectangular, both anterio- and posterio-lateral edge of paranota rounded, lacking projection; small projection starts on segment 4, then from 5 increasingly pointed, triangular, sublateral excavations on posterior margin becoming stronger. Lateral sides of paranota straight, outline of segments clearly delimited. Pore formula normal, pores in lateral central position on narrow paranota.

Segments 16–19 gradually tapering, posterior corners becoming obtuse, sublateral excavations disappear. Epiproct in dorsal view triangular, blunt, in lateral view slightly curved ventrad, with 4+4 large setae on tubercles on lateral sides, projection with 2+2 apical setae; paraprocts smooth, with two pairs of setae, upper ones on margin, lower ones on side; hypoproct semicircular with two setae on small tubercles.

Bases of midbody leg pairs weakly separated, sterna smooth and wide, pro- and metasterna fused. Coxa short, rectangular; prefemur ~ 2× as long as wide, with well-developed ventral spine; femur 1.5× longer than prefemur, incrassate; postfemur small, sub-globose; tibia slender, approximately same length as postfemur, tarsus 2× as long as tibia, slender, tapering towards small, curved claw.

Colour of living specimens (Fig. 16F) uniformly yellowish brown, only epiproct darker. Head, antennae, legs, and whole ventral side pale yellowish-greyish. Sides of collum and all paranota (except segment 19) with strong, pinkish-orangish spots.

**Figure 8.**
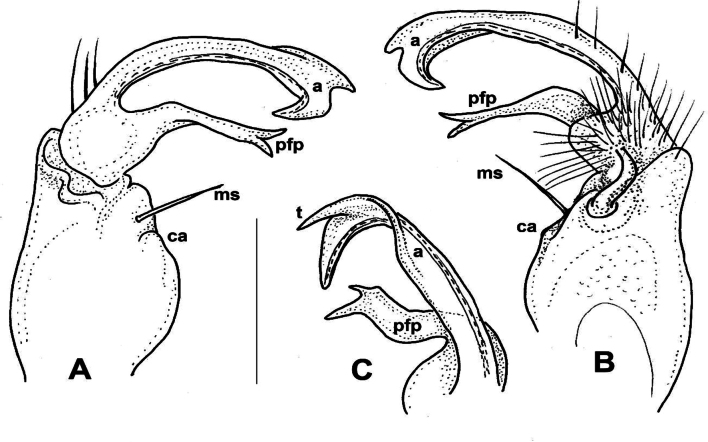
*Xystodesmussesokoensis* sp. nov., left gonopod of male paratype from Sesoko-jima Isl., Okinawa Pref. **A** lateral view **B** mesal view **C** Tip of telopodite, ventral view. Abbreviations: a = acropodite, ca = coxal apophysis, ms = macroseta, pfp = prefemoral process, t = tooth. Scale bar: 0.5 mm.

**Male characters**. Second leg pair with small coxal processes provided with three or four strong setae (as in *X.parvus* sp. nov., Fig. 6D), sterna and coxae of further legs without any modifications. Gonopods (Fig. 8A–C): Coxa stout, sub-globose, approximately as long as wide, instead of coxal apophysis only a small bump, with strong apophyseal macroseta (*ms*) above. Prefemur short, rectangular, densely setose on ventral side, with a few setae on dorsal side; prefemoral process (*pfp*) slender, shorter than acropodite, bent ventrad and ending in two small projections; acropodite (*a*) slightly broader, longer, only slightly bending subparallel to prefemoral process, gradually thickening towards tip, ending in two small projections: one broader, leaf-like, strongly curved ventrad, the other one like a small tooth (*t*) (Fig. 8C). Prostatic groove runs along medio-ventral side of acropodite.

Female unknown.

#### Remarks.

Sesoko-jima is a small island (area less than 3 km^2^) very close to Okinawa-jima Isl., and it is somewhat surprising that it holds an endemic species of millipede. The species seems to be restricted to a small forest on the island.

#### Etymology.

Named after the type locality, Sesoko-jima Island, connected by bridge to the island of Okinawa-jima Isl.. Adjective, masculine.

### ﻿ Xystodesmus variatus

Taxon classificationAnimaliaPolydesmidaXystodesmidae

(Pocock, 1895) comb. nov.

4FA8A5A4-8595-5041-AFBA-574743ECDD69

**Figure 9.**
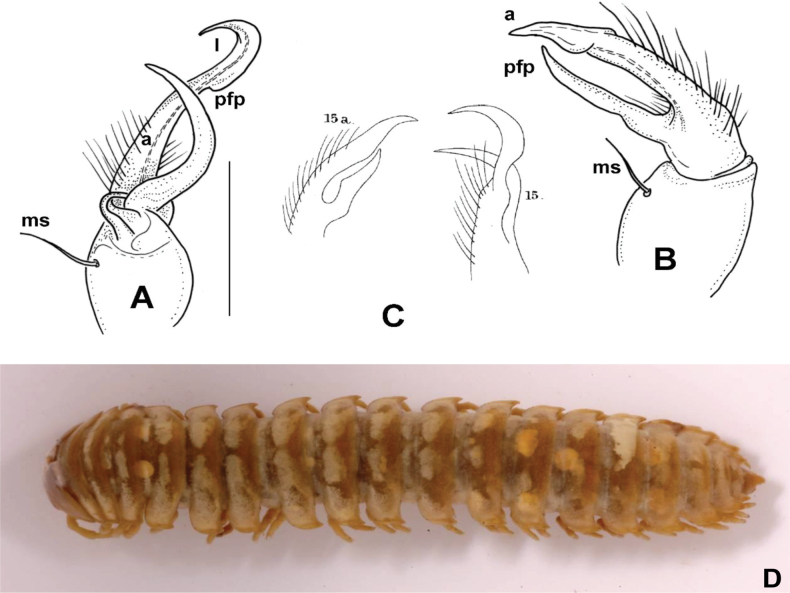
*Xystodesmusvariatus* (Pocock, 1895) comb. nov. **A, B** right gonopod of male from Kouri-jima Isl., Okinawa Pref., mesal and lateral views, respectively. **C** original figures by Pocock (1895), “fig. 15. *Fontariavariata*. Left copulatory foot from below, fig. 15a. Ditto, outer view.” **D***Fontariavariata* Pocock, 1895, holotype male specimen (BMNH, Reg. Nr. 1892.10.10.51). Abbreviations: a = acropodite, ms = macroseta, l = lobe, pfp = prefemoral process. Scale bar: 1 mm (**A, B**).

Figs 9A–9 , 4D , 17A, B

Fontaria variata Pocock, 1895: 361, figs 15, 15a; “Great Loo-Choo (Holst Coll.)” (= Okinawa)
Rhysodesmus variatus : Takakuwa 1954: 62
Riukiaria variata : Hoffman 1949: 5; Shinohara 1977: 118 (listed)
Riukiaria variata : Tanabe and Shinohara 1996: 1487 (“species possibly belonging to Xystodesmus”)
Riukiaria variata : Marek et al. 2014: 78

#### Type material examined.

***Holotype*** (Fig. 9D 39): • male, labeled as: “1892.10.10.51, Gr. Loo-Choo, purchased of H. Seebohm” (NHMUK).

#### Additional material examined.

• 1 male, Japan, Central Ryukyus, Okinawa Pref., Okinawa-jima Isl., Nakijin-son, Nakijin Village, 30 January 1996, leg. K. Yahata (NSMT-My 547); • 2 males, Japan, Central Ryukyus, Okinawa Pref., Okinawa Group, Okinawa-jima Isl., Ogimi Village, limestone hill near Mt. Nekumachiji-dake, 26°41.0'N, 128°08.1'E, 250 m a.s.l., 24 January 2009, leg. R., P., and Z. Korsós (HNHM diplo-04547); • 1 male, 4 females, Japan, Central Ryukyus, Okinawa Pref., Okinawa Group, Kouri-jima Isl., limestone hill, 26°42'08.6"N, 128°00'38.5"E, 40 m a.s.l., 30 January 2010, leg. R., P., and Z. Korsós (RUMF-ZD-00937); • 2 males, 3 females, Japan, Central Ryukyus, Okinawa Pref., Okinawa Group, Kouri-jima Isl., limestone hill, 26°42'08.6"N, 128°00'38.5"E, 50 m a.s.l., 18 April 2010, leg. Z. Korsós (1 female HNHM diplo-04548; 1 male, 1 female VMNH; 1 male, 1 female NHMD 1184734).

#### Diagnosis.

Small *Xystodesmus* with typical colour pattern still visible on preserved specimens, metaterga darker, proterga and paranota paler. Gonopod has only two branches, similar to *X.parvus* sp. nov. and *X.rebekae* sp. nov., but *X.parvus* sp. nov. is much smaller, and in *X.rebekae* sp. nov. both prefemoral process and acropodite are slender and straight, whereas in *X.variatus* comb. nov. they are strongly pointed and sickle-shaped in mesal view.

#### Description.

Measurements: length 29 mm, midbody segment width: 6.2 mm, metatergal length 2 mm; collum width 4.7 mm, median collum length 2.2 mm. Measurements of new material: length 24–29 mm, midbody segment width: 5.4–6.0 mm, metatergal length 1.2–1.4 mm; collum width 4.2–4.7 mm, median collum length 1.6–1.9 mm. Body sides between segments 5–15 parallel.

Head smooth, epicranial suture distinct. Antennal articles slightly clavate, first article sub-globose, articles 2–6 subequal in length, article 7 as long as wide.

Pro- and metaterga smooth, transverse depression on metaterga hardly noticeable. Collum in dorsal view elongated sub-hexagonal, with weak marginal ridges all around, lateral corners directed posterio-ventrad, weakly pointed, posterior margin wavy. Anterior edges of all paranota rounded, posterio-lateral corners increasingly pointed caudad, from segment 5 onwards with a concave posterior excision. Lateral sides of paranota arched, those bearing pores depressed, pore formula normal.

Segments 15–19 gradually tapering, caudal corners becoming more pointed. Epiproct protruding, in lateral view slightly curved, slender, with 2+2 apical and 4+4 lateral setae all on strong tubercles; paraprocts smooth, with two pairs of setae, upper ones on margins, lower ones on sides; hypoproct semicircular with 1+1 setae on small tubercles.

Bases of midbody leg pairs well separated (by 1.0–1.2 mm in male, 1.5–1.7 mm in females), sterna smooth and wide, pro- and metasterna fused together. Coxa ~ 2× as long as wide; prefemur ~ 1.5× longer, with well-developed ventral spine; femur 1.5× longer than coxa, clavate; postfemur shorter than coxa, approximately as long as wide, tibia and tarsus slender, tarsus ~ 1.5× longer than tibia; claw small, slightly curved ventrad.

Colour of newly collected living specimens (Fig. 17A, B) generally greyish, blueish brown with orange or yellowish paranotal spots. These are less conspicuous than in other *Xystodesmus* species (e.g., in *X.kumamotoensis* sp. nov. or *X.rebekae* sp. nov.). Proterga and sides of metaterga paler, more greyish, and in most cases a dark dorsomedial line also traceable, at least in midbody region. Preserved specimens lost colouration, but differences between pro- and metaterga, and the metatergal side spots still visible. Clypeus and epiproct pale brownish, underside of head, antennae, legs, and whole ventral side pale whitish.

**Male sexual characters**. Second leg pair with small coxal processes provided with three or four strong setae (as in *X.parvus* sp. nov., Fig. 6D), sterna of segments 4–6 and further legs without any modifications. Gonopods (Fig. 9A–C): Coxa stout, approximately as long as wide, coxal apophysis completely lacking, apophyseal macroseta (*ms*) relatively small. Prefemur short, sub-globose, densely setose on ventral side, setosity goes on to halfway of acropodite; prefemoral process (*pfp*) slender, curved ventrad, ~ ¾ as long as acropodite, ending in a long, pointed tip; acropodite (*a*) slightly broader at base, gradually tapering towards pointed tip, but subapically with a leaf-like broadening (*l*), in mesal view similar to an eagle’s claw (Fig. 9A). Prostatic groove runs along the mesal side of acropodite to the pointed tip.

**Figure 10.**
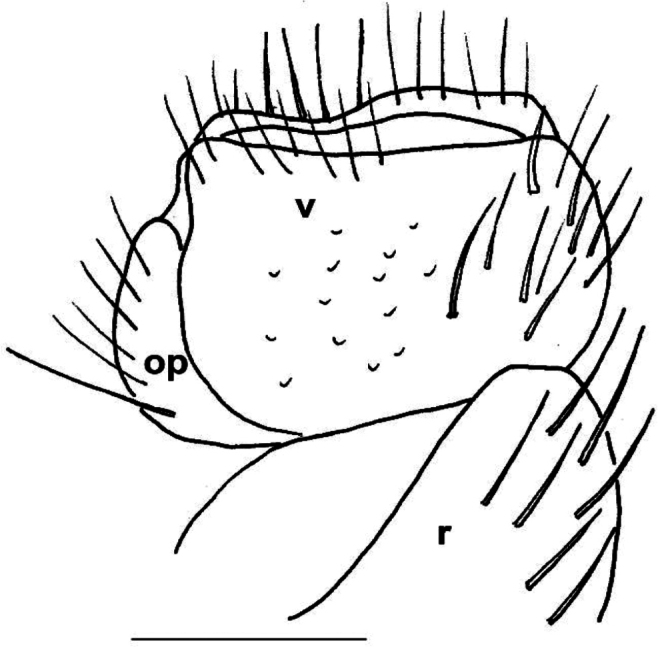
*Xystodesmusvariatus* (Pocock, 1895), comb. nov. Left cyphopod of female from Kouri-jima Isl., Okinawa Pref., anterior view. Abbreviations: op = operculum, r = receptacle, v = valve. Scale bar 0.5 mm.

**Female sexual characters**. Cyphopods (Fig. 10) deeply embedded in joint aperture closely behind leg pair 2, encapsulated in separate vulval sacs. Receptacula (*r*) on both anterior and posterior side, low, subtriangular, setose on entire surface; operculum (*op*) narrow, ~ ¾ as high as bursal valves; valves (*v*) rectangular, laterally with small projected tips, with several row of short setae along margins.

#### Remarks.

With the re-examination and the side-by-side comparison of the type specimens of *Fontariavariata* Pocock, 1895 and the freshly acquired specimens it became clear that they were conspecific. In the original description the colour, obviously based on preserved specimens, is described as “upper surface rather thickly clouded with fuscous, with a clearer spot on each side above the keels” (Pocock 1895: 361); and those spots correspond to the orange paranotal spots observed on the living specimens. The body size, the shape and structure of metaterga and paranota, and especially the gonopods are in complete agreement in both samples, so the assignment of *F.variata* to *Xystodesmus* is justified.

Hoffman (1949) placed *F.variata* in *Riukiaria* together with *F.holstii* Pocock, 1895, but Tanabe and Shinohara (1996) tentatively separated them in different genera, assigning *variatus* “possibly” to *Xystodesmus*. In the last part of their paragraph, Tanabe and Shinohara (1996: 1487), perhaps due to a typographical error, referred to *variatus* together with other species described by Takakuwa, saying that “*Rhysodesmusspinosissumus* (recte *spinosissimus*), *Riukiariageniculata*, *Ri.spiralipes*, *Ri.variata*, *K.amoea* (recte *amoena*), and *Pachydesmusbazanensis*, their types, which had been deposited in Y. Miyosi’s private collection, were destroyed in 1945”. However, *R.variata* was actually described by Pocock (1895), not Takakuwa, and its type has never been in Miyosi’s private collection, but has been stored and was recently found in the Natural History Museum, London, and is redescribed. The newly collected specimens were found in association with *Riukiariaholstii* (Pocock, 1895), a widespread species on the central and northern part of Okinawa-jima Island. This long-known species has also been recorded from more southwestern and remote islands in the Ryukyu Archipelago, such as Ishigaki-jima and Iriomote-jima in the Yaeyama Group and Uotsuri-jima in the Senkaku Group (Nakamura and Korsós 2010). However, from our extensive myriapodological surveys conducted in the archipelago, we can say with near certainty that these records should be regarded as misidentifications. There are superficially similar species such as *Riukiariachelifera* (Takakuwa, 1941), endemic to the Yaeyama Group. The “*Rhysodesmusvariatus*” found on Uotsuri-jima Island (Ikehara and Shimojana 1971) may be an undescribed species due to the biogeographic peculiarities of the terrestrial faunas in the Senkaku Group among Japanese islands (Ota 1998; Kurita et al. 2017).

### ﻿ Xystodesmus pallidus

Taxon classificationAnimaliaPolydesmidaXystodesmidae

(Verhoeff, 1937) comb. nov.

60AB2267-ECD1-58F3-BE88-A3C33DCD25AE

**Figure 11.**
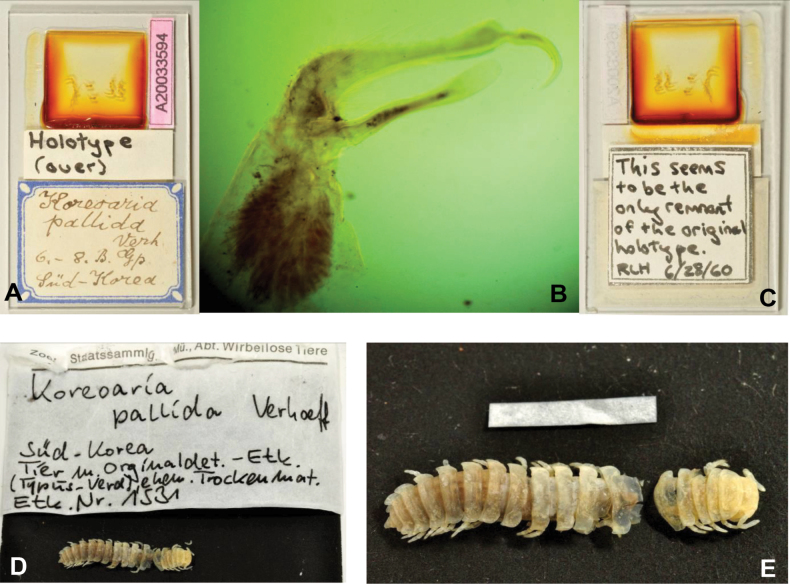
*Xystodesmuspallidus* (Verhoeff, 1937), comb. nov. **A–C** Slide of male gonopods and 6–8^th^ leg pairs of holotype from “Süd-Korea” (ZSMC, Reg. Nr. A20033594) **B** right gonopod in slide, lateral view **C** back of slide with comment by R. L. Hoffman, 28 June 1960 **D, E** male holotype in 2 pieces (ZSMC, Reg.Nr. A20060019). Scale bar: 10 mm (**E**).

**Figure 12.**
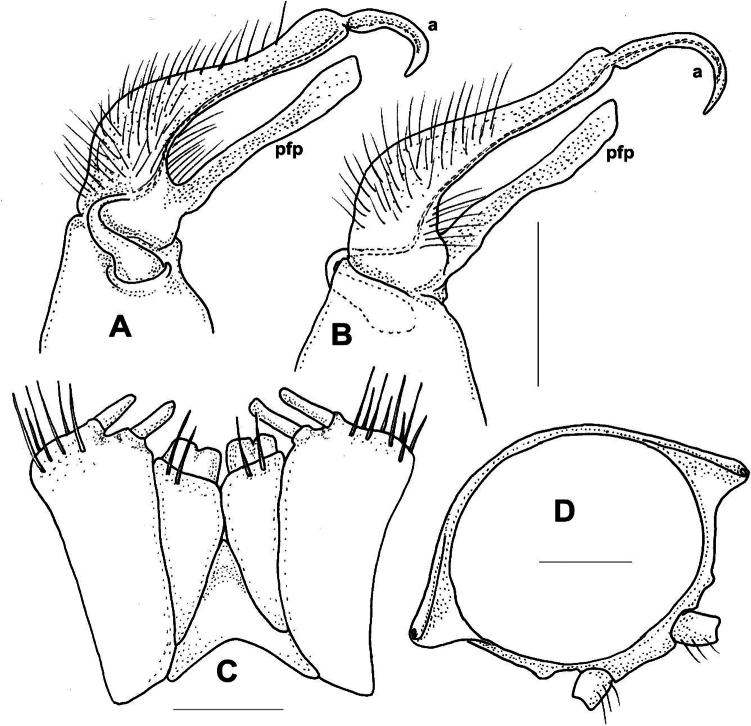
*Xystodesmuspallidus* (Verhoeff, 1937), comb. nov. **A, B** right and left gonopods of holotype male, drawn from slide (Fig. 11B), mesal and lateral views, respectively **C** gnathochilarium of male holotype, ventral view **D** cross section of broken female paratype, 5^th^ segment, posterior view. Abbreviations: a = acropodite, pfp = prefemoral process. Scale bars: 0.5 mm (**A–C**); 1 mm (**D**).

**Figure 13.**
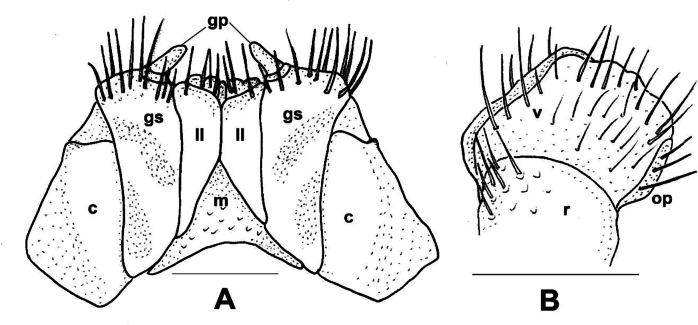
*Xystodesmuspallidus* (Verhoeff, 1937), comb. nov. **A** gnathochilarium of female paratype, ventral view **B** left vulva of female paratype, posterior view. Abbreviations: c = cardo, gp = gnathochilarial palps, gs = gnathochilarial stipites, ll = lingual lobes, m = mentum, op = operculum, r = receptacle, v = valve. Scale bars: 0.5 mm.

Figs 11A–E , 12A–D , 13A, B

Koreoaria pallida Verhoeff, 1937: South Korea (Verhoeff 1937: 319–320, fig. 8)
Koreoaria pallida : Tanabe and Shinohara 1996: 1487 (“species possibly belonging to Xystodesmus”)
Koreoaria pallida : Tanabe and Shinohara 1996: 1487
Koreoaria pallida : Marek et al. 2014: 71

#### Type material examined.

***Holotype***: • male (ZSMC, Reg. Nr. A20060019): in 2 fragments, gonopods and legs are on separate slide (ZSMC, Reg. Nr. A20033594) (Fig. 11). ***Paratypes***: • 4 females (ZSMC, Reg. Nr. A20060019).

#### Description of holotype.

Male total body length ~ 21 mm; collum length 1.5 mm, width 3.3 mm; 10^th^ segment width 4.2 mm, metazonal length 1.1 mm. Paranotal processes from segment 6 onwards projected caudad, anterior margin broadly rounded, male 2^nd^ leg pair with long, slender coxal projection, 3^rd^ with nothing, 4^th^ and 5^th^ with small, triangular coxal processes; postgonopodal prefemoral spines strong and long, half as long as length of prefemur, sternal plate broad, coxal distance 1.2 mm, anterior-posterior sterna fused.

***Male gonopods*** (Figs 11A–C, 12A, B) fits well to the original description and drawing.

***Female paratypes***. Total body length 22–26 mm; collum length 1.6–1.8 mm, width 3.5–3.8 mm; 10^th^ segment paranotal width 4.4–5.1 mm, metazonal length 1.1–1.2 mm, coxal distance 1.1–1.5 mm. Gnathochilarium (Figs 12C, 13A) and cyphopods (Fig. 13B) are here illustrated for the first time.

***Colouration*** (Fig. 11D–E). Type material in alcohol completely faded. Verhoeff (1937: 319), however, provided a description which is in total agreement of *Xystodesmus* characters: “braune Metatergite und rötlichgelben Längswisch auf den Seitenflügeln” [metatergites brown, and paranota with reddish yellow longitudinal spots].

#### Remarks.

The flat gonopodal prefemoral process (*pfp*, Fig. 12A, B) was called by Verhoeff (1937: 317, fig. 8) as “abgespaltener Tibiotarsus”, and in the generic comparison of *Koreoaria* he had set it against the prefemoral process of *Pachydesmus* Cook, 1895. According to Verhoeff, the longer process made of two parts, the “praefemorofemur” and its disjunct ending, the solenomere (Verhoeff 1937: fig. 8, *sl*). In our opinion this is a misunderstanding, as the longer process corresponds to the acropodite (Fig. 12A, B, *a*), and the other one is the prefemoral process (correctly interpreted by Takakuwa 1942b for *Koreoariaamoena*).

### ﻿ Xystodesmus amoenus

Taxon classificationAnimaliaPolydesmidaXystodesmidae

(Takakuwa, 1942) comb. nov.

D02F6C3E-8795-55D0-B9F6-09E3FF2C5330

Fig. 15A

Koreoaria amoena Takakuwa, 1942b: Daegu, South Korea (Takakuwa 1942b: 362–363, fig. 5)
Koreoaria amoea (sic!): Tanabe and Shinohara 1996: 1487: (“species possibly belonging to Xystodesmus”)
Koreoaria amoea (sic!): Marek et al. 2014: 71

#### Descriptive notes

based on Takakuwa (1942b: 366). Body length 22 mm, width 4 mm. Male gonopod with two processes (Fig. 15A, redrawn from Takakuwa 1942b:. 362, fig. 5), the femoral process continues in tibiotarsus (*a*) with a small tooth (*b*), taking the seminal groove all along to the end bending backwards (acropodite); whereas the other shorter and thinner process is the prefemoral process (“Prefemoralfortsatz”).

#### Colouration.

Without the type material and fresh specimens, we can only rely on Takakuwa’s description (1942b: 366): “Rücken graulich, Kopf, Antennen und Bauch gelb, Bein proximal etwas graulich, distal gelb. Seitenflügel mit einem deutlich rötlichen Fleck.” [Back greyish, head, antennae, and belly yellow, legs proximally somewhat greyish, distally yellow. Paranota with a clearly reddish spot.] The important part is the last sentence, which is again in total agreement of *Xystodesmus* characters.

#### Remarks.

Type material could not be studied since Takakuwa’s private collection, including type material of many species, is considered lost (Tanabe and Shinohara 1996; Chao and Chang 2008).

### ﻿ Xystodesmus saltuosus

Taxon classificationAnimaliaPolydesmidaXystodesmidae

(Haga, 1968) comb. nov.

5976EB48-6298-525C-A660-9016AE4DBB9F

Fig. 14A–C

Rhysodesmus saltuosus Haga, 1968: 8, fig. 11a–f: Mt Sarakura-yama, Kita-Kyushu City, 18 June 1961, leg. A. Haga

#### Type material examined.

Holotype male (NSMT-My.162).

#### Redescription.

Length not possible to measure (holotype is in 5 pieces), but apparently under 30 mm, midbody width with paraterga 4.8 mm, midbody metatergal length 1.2 mm, collum width 4 mm, median collum length 1.9 mm. Body sides between segments 7–14 subparallel.

Head smooth, with two frontal setae, epicranial suture distinct. Antennal articles slightly clavate, first article sub-globose, articles 2–6 subequal in length, article 7 small, as long as wide.

Collum in dorsal view subtrapezoidal, no traces of marginal ridges, lateral corners rounded, directed posterio-ventrad, posterior margin slightly wavy. Pro- and metaterga smooth, transverse depression on metaterga hardly noticeable. Anterior edges of all paranota rounded, posterio-lateral corners increasingly pointed caudad, from segment 5 onwards with a concave posterior excision. Lateral sides of paranota arched, those bearing pores depressed, pore formula normal.

Segments 15–19 gradually tapering, caudal corners increasingly pointed. Epiproct in dorsal view triangular, in lateral view slightly curved ventrad, with 2+2 apical and 4+4 lateral setae all on strong tubercles; paraprocts smooth, with two pairs of setae, upper ones on margins, lower ones on sides; hypoproct semicircular with 1+1 setae on small tubercles.

Bases of midbody leg pairs well separated by 1.0 mm, sterna smooth and wide, pro- and metasterna well separated. Coxa sub-globose; prefemur ~ 1.2× longer, with well-developed ventral spine on postgonopodal legs; femur incrassate, ~ 2× as long as wide; postfemur much shorter, approximately as long as wide, tibia and tarsus slender, subequal in length, ~ 2× as long as wide; claw large, flat, curved ventrad.

Colour of living specimens unknown; holotype completely faded due to preservation, currently yellow, and even pro- and metatergal differences cannot be traced.

**Figure 14.**
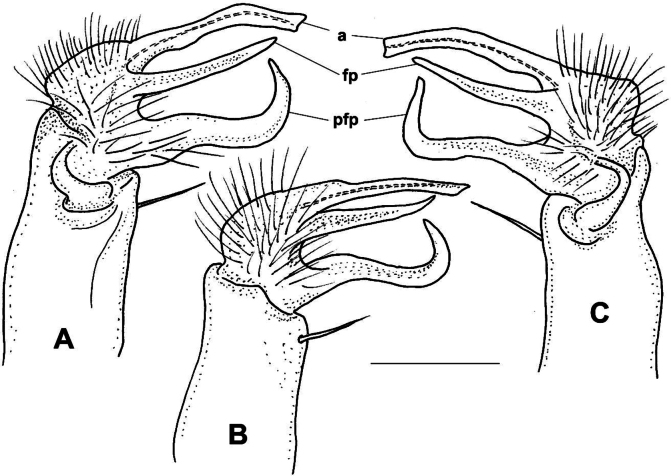
*Xystodesmussaltuosus* (Haga, 1968), comb. nov., holotype male from Kita-Kyushu (NSMT-My.162) **A** right gonopod mesal view **B, C** left gonopod lateral and mesal views, respectively. Abbreviations: a = acropodite, fp = femoral process, pfp = prefemoral process. Scale bar: 0.5 mm.

**Male sexual characters**. Second leg pair with small coxal processes with a pair of strong setae (as in *X.rebekae* sp. nov., Fig. 7C), sterna of segments 4–6 and leg pairs 3 onwards without any modification. Gonopod (Fig. 14A–C) coxa long and slender, ~ 2× as long as wide, coxal apophysis completely lacking, apophyseal macroseta relatively small. Telopodite built of three subequal, subparallel processes: prefemoral process (*pfp*) sits on short, densely setose prefemur; approximately as long as acropodite, curved distad at its end; solenomere has two processes, acropodite (*a*) subparallel-sided, flattened, tip rectangular as if cut off, prostatic groove runs along mesal side; and at its base, from the femoral part a third process (*fp*) starts, slightly shorter than acropodite, gradually tapering towards long, pointed tip.

**Figure 15.**
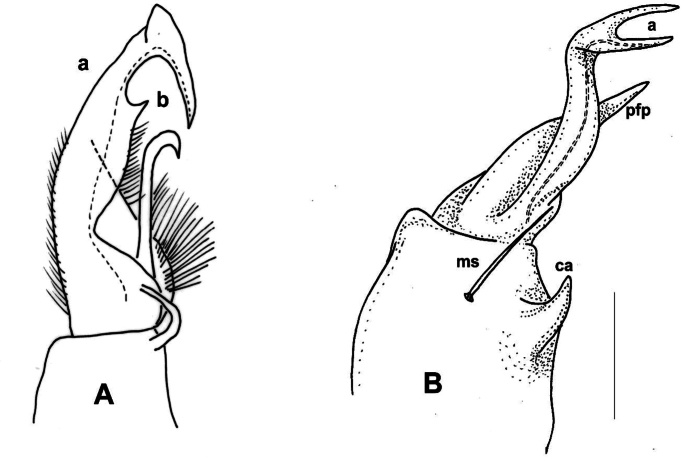
Male gonopods of *Xystodesmus* species. **A***Xystodesmusamoenus* (Takakuwa, 1942), comb. nov., right gonopod of male type specimen from Daegu (= Taikyu), South Korea, redrawn from Takakuwa’s original (1942b: 362, fig. 5), mesal view, not to scale. Abbreviations: “a = Tibiotarsus, b = Femurabschnitt mit kleiner Spitze” **B***Xystodesmusyamamiensis* Masuda, 2001, left gonopod of holotype male from Yamami, Aichi Pref., Japan (NSMT-My.294), lateral view. Abbreviations: a = acropodite, ca = coxal apophysis, ms = macroseta, pfp = prefemoral process. Scale bar: 0.5 mm (**B**).

Female unknown.

#### Remarks.

*Rhysodesmus* is a Middle American genus, and the name was mistakenly used by early Japanese authors, mainly following the prominent Japanese myriapodologist Yoshioki Takakuwa (1873–1960). The situation was first explained in Japanese by Shinohara (1977), but also established by Hoffman (1980), Marek et al. (2014), and Huerta-de la Barrera et al. (2021). The species itself (*Rh.saltuosus*) was unfortunately overlooked by Shinohara (1977), Tanabe and Shinohara (1996), and Marek et al. (2014) since it was published in a private but widely available publication (Haga 1968). Huerta-de la Barrera et al. (2021) referred to Haga’s publication, but did not specifically mentioned *Rh.saltuosus.* Since the holotype male was available for study we could establish its association with *Xystodesmus* and removed the species from *Rhysodesmus*.

### ﻿ Xystodesmus martensii

Taxon classificationAnimaliaPolydesmidaXystodesmidae

(Peters, 1864)

D80AF95E-F811-5206-B688-EE9D2BED4D32

Fig. 17C, D

Polydesmus (Fontaria) martensii Peters, 1864: 531, no fig.
Xystodesmus martensii : Cook 1895: 5, no fig.

#### Material examined.

• 4 males, 3 females, Japan, Honshu, Kanagawa Pref., Fujisawa City, Eno-shima Isl., Kodama Shrine, 35°18'03"N, 139°28'52"E, 20 m a.s.l., oak grove, 11 October 2010, leg. Z. Korsós and Y. Nakamura (HNHM diplo-04549); • 3 males, 4 females, Japan, Honshu, Kanagawa Pref., Isehara City, Mt. Oyama, Yabitsu Pass, 760 m a.s.l., 35°25'36"N, 139°14'05"E, *Cryptomeriajaponica* forest, 11 October 2010, leg. Z. Korsós and Y. Nakamura (HNHM diplo-04550); 1 male, Japan, Honshu, Kanagawa Pref., Yamakita Town, Yuzuku, Myojin Pass, 12 October 2010, leg. Y. Nakamura (RUMF-ZD-00935)

#### Descriptive notes.

Tanabe and Shinohara (1996) gave an extensive redescription of the species based on their new material and have shown great variation in the shape of male gonopods. The gonopods of the eight males we studied here fit within the range of variation described by Tanabe and Shinohara (1996). We also noticed the conspicuous metatergal tubercles, which are much more developed than in other *Xystodesmus* species.

#### Colour of living specimens

(Fig. 17C, D). The Kanagawa samples represent two slightly different colour morphs: one pale greyish, the other one dark brown, but both have the typical and strong, reddish orange spots on paranota.

### ﻿ Xystodesmus nikkoensis

Taxon classificationAnimaliaPolydesmidaXystodesmidae

(Chamberlin & Wang, 1953)

59336AE4-CC57-597A-ACD0-65B8052FF9A1

Fig. 17E, F

Nikkonus nikkoensis Chamberlin & Wang, 1953: 9, fig. 3
Xystodesmus nikkoensis : Tanabe and Shinohara 1996: 1484, figs 3–7, 11J–K, 13A, 16A, 17L, M

#### Material examined.

• 4 males, Japan, Northern Ryukyus, Kagoshima Pref., Osumi Group, Yaku-shima Isl., Anbo forest road, *Cryptomeriajaponica* forest, 30°18'42.0"N, 130°37'54.0"E, 190 m a.s.l., 5 July 2010, leg. Z. Korsós (HNHM diplo-04551).

#### Descriptive notes.

Tanabe and Shinohara (1996) gave a short redescription and showed relatively small variability in the male gonopod structure. Our specimens, collected exactly on the original type locality (“Anbo Rindo”, Yaku-shima Island) are in complete agreement. Tanabe and Shinohara (1996, p. 1484) also said *X.nikkoensis* is “the smallest species of xystodesmid in Japan”. This statement, however, in light of the present discovery of the new species *X.parvus* sp. nov. and *X.sesokoensis* sp. nov., is not true anymore.

#### Colour of living specimens

(Fig. 17E, F). According to Tanabe and Shinohara (1996: 1484) Yaku-shima specimens have on “collum and metatergites with yellowish white paranotal spots”. Based on our freshly photographed animals also from Yaku-shima Isl., we can support this observation and can add that those paranotal spots are even more conspicuous next to the entirely dark brown body colour.

### ﻿ Xystodesmus yamamiensis

Taxon classificationAnimaliaPolydesmidaXystodesmidae

Masuda, 2001

167C9B1E-4A2E-5C2D-A1CE-C8878F50C5CE

Fig. 15B

#### Material examined.

***Holotype*** male (NSMT-My.294), and ***paratype*** male (NSMT-My. 295), Aichi Pref., Chita County, Minamichita Town, Yamami, 5 April 1998, leg. K. Masuda.

#### Descriptive notes.

The original description and illustrations lack some important details, thus after the re-examination of the holotype, we present here an additional drawing of the male gonopod (Fig. 15B). Coxa stout, as wide as long, with a strong apophyseal process (*ca*) and a large macroseta (*ms*). Prefemur and acropodite slightly twisted, prefemoral process (*pfp*) ~ ¾ of length of acropodite, those of two gonopods crossing each other *in situ*. Prostatic groove runs along lateral side of acropodite (*a*) and ends on distal part of lower branch of bifurcated tip.

#### Remarks.

Interestingly, the gonopods have a general similarity to *Riukiarianeptuna* (Pocock, 1895) (Korsós et al. 2011), as the end of acropodite is ramified: two branches in *X.yamamiensis*, three in *R.neptuna* (see Pocock 1895; Hoffman 1949). However, the body size (19–24 mm) and especially the colour of *X.yamamiensis* (“Keels have a red spot in roughly in the middle.” Masuda 2001: 635) clearly indicate that this is a species of *Xystodesmus*.

**Unidentified *Xystodesmus* female 1**

Fig. 16G

**Material examined**. • 6 females, Japan, Central Ryukyus, Okinawa Pref., Okinawa Group, Tokashiki-jima Isl., National Okinawa Youth Fellowship Center, mixed forest, 26°12'40.7"N, 127°21'46.4"E, 200 m, 9–10 April 2010, leg. Z. Korsós (No. 183) (RUMF); • 2 females, Japan, Central Ryukyus, Okinawa Pref., Okinawa Group, Tokashiki-jima Isl., Tokashiki, Otani forest road, mixed forest, 26°12'45.3"N, 127°21'26.3"E, 18 m, 10 April 2010, leg. Z. Korsós (HNHM diplo-04552); • 2 females, Japan, Central Ryukyus, Okinawa Pref., Okinawa Group, Tokashiki-jima Isl., National Okinawa Youth Fellowship Center, outside the fence, mixed forest, 26°12'44"N, 127°21'50"E, 215 m, 21 October 2010, leg. Z. Korsós (No. 266) (RUMF)

**Figure 16.**
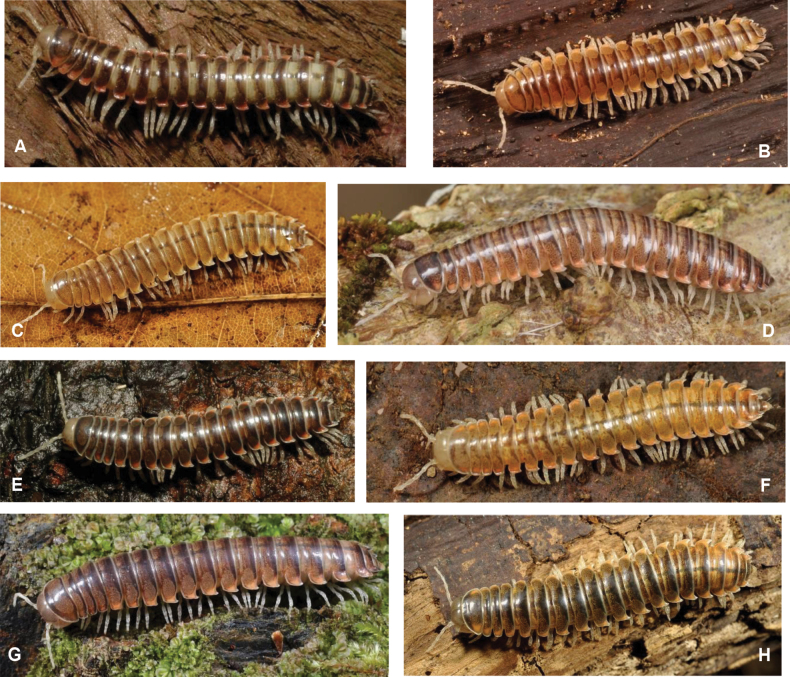
Live habitus of new *Xystodesmus* millipede species **A***X.fasciatus* sp. nov., male paratype, Fukiage, Kagoshima Pref. **B***X.keramae* sp. nov., male paratype, Aka-jima Isl., Okinawa Pref. **C***X.kumeensis* sp. nov., male paratype, Kume-jima Isl., Okinawa Pref. **D***X.parvus* sp. nov., male paratype, Okinoerabu-jima Isl., Kagoshima Pref. **E***X.rebekae* sp. nov., male paratype, Mt. Yae-dake, Okinawa-jima Isl., Okinawa Pref. **F***X.sesokoensis* sp. nov., male holotype, Sesoko-jima Isl., Okinawa Pref. **G** unidentified female, Tokashiki-jima Isl., Okinawa Pref. **H** unidentified female, Tokuno-shima Isl., Kagoshima Pref. Not to scale.

**Colour of living specimens** (Fig. 16G). Body uniformly dark brown, posterior edge of metaterga darker; paranota from 5^th^ segment onward light, yellowish orange; head brown, antennae, legs, and underside whitish.

**Figure 17.**
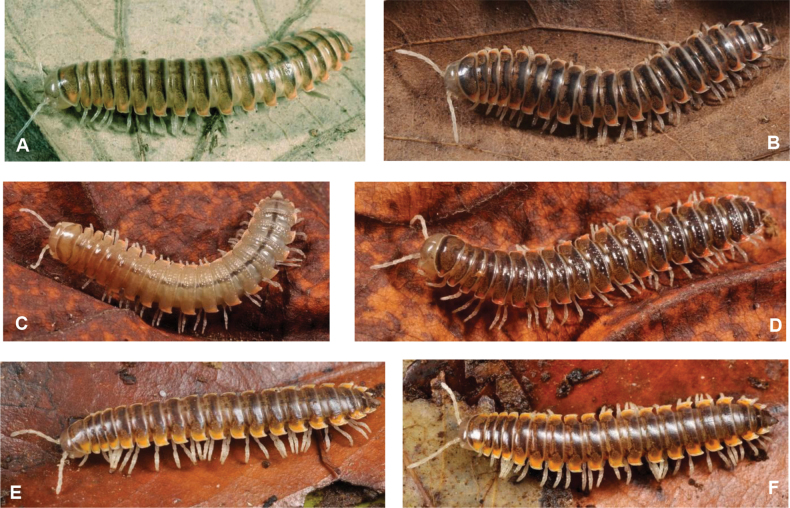
Live habitus of *Xystodesmus* millipedes **A, B***X.variatus* (Pocock, 1895) males, Kouri-jima Isl., Okinawa Pref. **C, D***X.martensii* (Peters, 1864) males **C** Eno-shima Isl., Kanagawa Pref. **D** Mt. Oyama, Kanagawa Pref. **E, F***X.nikkoensis* (Chamberlin & Wang, 1953) males, Yaku-shima Isl., Kagoshima Pref. Not to scale.

**Remark**. Probably the same species as the one occurring on Aka-jima Isl., i.e., *X.keramae* sp. nov. (Fig. 16B).

**Unidentified *Xystodesmus* female 2**

Fig. 16H

**Material examined**. • 1 female, Japan, Central Ryukyus, Amami Group, Tokuno-shima Island, Kametsu, near the pass between Mt. Inokawa-dake and Mt. Hage-dake, 27°45'38.4"N, 128°58'53.7"E, *Castanopsissieboldii* forest, 4 February 2012, leg. Y. Nakamura (HNHM diplo-04553)

**Colour of living specimens** (Fig. 16H). Body uniformly dark greyish brown, median and posterior part of metaterga darker; all paranota including collum with light, yellowish spots; head and epiproct greyish brown; antennae, legs, and underside whitish.

**Remark**. Up to now this is the only *Xystodesmus* specimen from Tokuno-shima Island and probably represent a different species. However, without knowing the male it would be premature to assign it to any category.

### ﻿Key to the species of *Xystodesmus*

**Figure 18.**
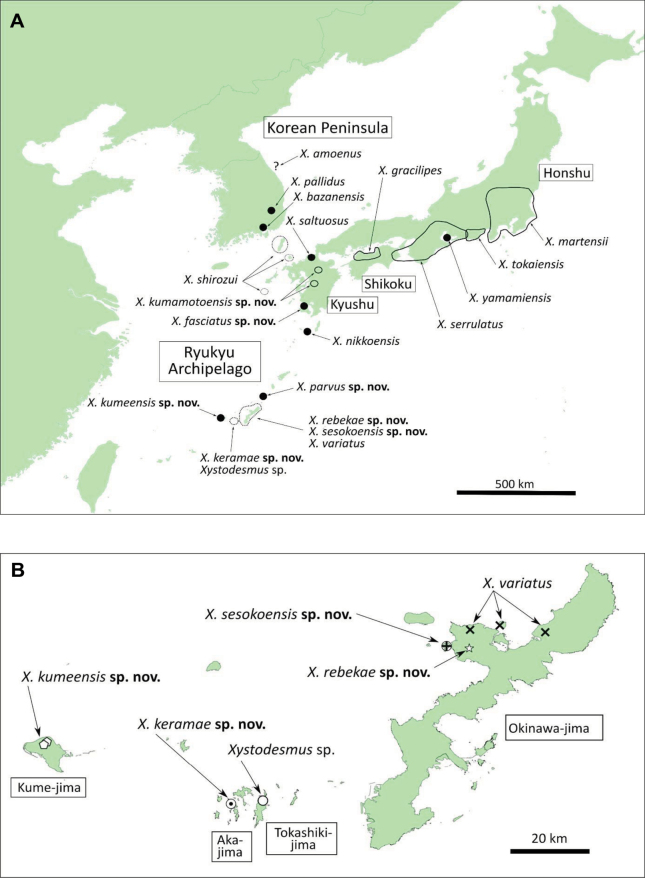
Distribution maps of *Xystodesmus* species dealt in this paper **A** map of Japan (Honshu, Shikoku, Kyushu, the Ryukyu Archipelago) and the Korean Peninsula, where species of *Xystodesmus* occur **B** map of the Okinawa Island Group with the occurrence of five *Xystodesmus* species.

Tanabe and Shinohara (1996) already provided a tentative key to the six species of *Xystodesmus* which they accepted as valid at that time (*X.shirozui*, *X.martensii*, *X.gracilipes*, *X.nikkoensis*, *X.serrulatus*, and *X.tokaiensis*). Since then, one new species, *X.yamamiensis* was described in 2001, so including the four new combinations and seven new species presented in this paper, we have now 18 *Xystodesmus* species for which to construct a complete key. This is a difficult task, and we agree with Tanabe and Shinohara (1996) that because of the significant variation in gonopodal morphology, a comparison of the illustrations and reference to the distribution will sometimes be more efficient. The key below starts with the countries (Korea and Japan, see also the maps in Fig. 18A, B), then body size (total length and maximum midbody width), but later only male gonopodal characters, and sometimes colouration, were used to separate the species.

**Table:** 

1	Species from the Korean Peninsula	**2**
–	Species from the Japanese islands	**3**
2	Male gonopod with 2 simple, forceps-like processes; acropodite with curved, thin solenomere, prefemoral process parallel-sided (Figs 11B, C, 12A, B); 4 ^th^ and 5^th^ legs with small, triangular coxal processes (Korean Peninsula)	***X.pallidus* (Verhoeff, 1937), comb. nov.**
–	Male gonopod with strong, thick acropodite, solenomere bending at right angle, prefemoral process thin and curved at the end (Fig. 15A); no triangular coxal processes on 4^th^ and 5^th^ legs (Korean Peninsula)	***X.amoenus* (Takakuwa, 1942), comb. nov.**
3	Body length < 25–27 mm, midbody width < 5.0–5.2 mm	**4**
–	Body length ~ 30 mm, midbody width > 5.2, often above 6.0 mm	**7**
4	Male gonopod with strong coxal apophysis. End of acropodite bifurcated (Aichi Pref., Honshu)	***X.yamamiensis* Masuda, 2001**
–	Male gonopod without coxal apophysis, or only with 1 small bump	**5**
5	Male gonopod stout, with strong, tongue-like prefemoral process; acropodite with strong femoral process and 1 small tooth under its tip (Fig. 3A–C). (Kumamoto and Oita Pref., Kyushu)	***X.kumamotoensis* sp. nov.**
–	Male gonopod is different; prefemoral process slender, acropodite is simple, without additional processes	**6**
6	Coxa of male gonopod with 1 small bump (but not apophysis!); prefemoral process slender with 2 small branches at tip; acropodite long, bending, apex is thicker with 1 small tooth (Fig. 8A–C). Body colour yellowish brown with pale orange paranotal spots (Fig. 16F). (Sesoko-jima Island, Okinawa Pref.)	***X.sesokoensis* sp. nov.**
–	Coxa of male gonopod with no modifications; prefemoral process and acropodite similar in size, spiralling around each other, tip of solenomere leaf-like (Fig. 6A–C). Body colour pale brownish with pale orange paranotal spots (Fig. 16D). (Okinoerabu-jima Island, Kagoshima Pref.)	***X.parvus* sp. nov.**
7	Male gonopods with coxal apophysis	**8**
–	Male gonopods without coxal apophysis	**13**
8	Coxal apophysis of male gonopod very strong, almost hook-like; prefemoral process flat and wide, curving backwards; acropodite with 3 small teeth at tip (Fig. 1A, B). Colour pattern fasciated with grey prozona and dark brown metazona, paranotal spots strong reddish (Fig. 16A). (Kagoshima Pref., Kyushu)	***X.fasciatus* sp. nov.**
–	Coxal apophysis weaker, not hook-like; gonopod structure less complicated, usually with only 2 subequal processes	**9**
9	Gonopods with a cup-shaped acropodite process (see Tanabe and Shinohara 1996: figs 12A, B, 14). Colouration greyish to dark brown, with strong red spots on paranota (Fig. 17C, D). Metaterga with 2 or 3 rows of conspicuous tubercles. (Ibaraki, Tochigi, Gunma, Saitama, Chiba, Tokyo, Kanagawa, Yamanashi and Shizuoka Pref., Honshu)	***X.martensii* (Peters, 1864)**
–	Gonopods different, without cup-shaped process; paranotal spots not so strong. Metaterga without strong tubercles	**10**
10	Coxal apophysis well developed; gonopods with 2 simple, parallel, subequal processes	**11**
–	Coxal apophysis weak, only slightly larger than a bump; gonopod processes always with some toothed or leaf-like extensions	**12**
11	Gonopod acropodite and prefemoral process subequal in length, very slender, entirely straight (see Tanabe and Shinohara 1996: figs 12D, 15D-I). (Aichi, Gifu, Nara, Mie, and Shizuoka Pref., Honshu, and Tokushima Pref., Shikoku)	***X.serrulatus* (Miyosi, 1952)**
–	Prefemoral process little longer than acropodite and curved backwards (Fig. 2A–C). Colouration pale brown, paranotal spots pale yellowish (Fig. 16B). (Aka-jima Island and Tokashiki-jima Isl., Okinawa Pref.)	***X.keramae* sp. nov.**
12	Prefemoral process shorter or subequal to acropodite; ventrally at midlength with a small triangular process (see Tanabe and Shinohara 1996: figs 13A, 16A). Colouration dark brown, with bright yellow paranotal edges (Fig. 17E, F). (Yaku-shima Isl., Kagoshima Pref.)	***X.nikkoensis* (Chamberlin & Wang, 1953)**
–	Prefemoral process longer than acropodite, twisted at the end, and with a tooth with wide base at its midlength; acropodite with a small lamella at tip (Figs 5A–C). Colouration pale yellowish, paranotal spots almost invisible (Fig. 16C). (Kume-jima Isl., Okinawa Pref.)	***X.kumeensis* sp. nov.**
13	Male gonopod with 3 processes (prefemoral, femoral, and acropodite)	**14**
–	Male gonopod with only 2 processes (prefemoral and acropodite)	**15**
14	Prefemoral process long, straight, and slender, with pointed tip; femoral process close to acropodite, latter with median flange (see Tanabe and Shinohara 1996: figs 13C, D, 16C, D). Tsushima Isl., Iki Isl., and Danjo Isl. (Oshima and Meshima) (Nagasaki Pref., Kyushu)	***X.shirozui* (Takakuwa, 1942)**
–	Prefemoral process curved, acropodite blunt, with a third femoral process arising near the base (Fig. 14A–C). (Fukuoka Pref., Kyushu)	***X.saltuosus* (Haga, 1968), comb. nov.**
15	Prefemoral process of gonopod much shorter than acropodite; the latter with long, needle-like median branch (see Tanabe and Shinohara 1996: fig. 13B) (Ehime Pref., Shikoku)	***X.gracilipes* (Takakuwa, 1943)**
–	Prefemoral process and acropodite subequal in length	**16**
16	Acropodite and prefemoral process very close to each other, parallel, wide, and sickle-shaped, strongly curved medially; latter sometimes with lateral or median branch (see Tanabe and Shinohara 1996: figs 12C, 15A-C). (Shizuoka Pref., Honshu)	***X.tokaiensis* Tanabe & Shinohara, 1996**
–	Prefemoral process and acropodite stand widely separated, without any branches, slender, straight, or only slightly curved	**17**
17	Prefemoral process large, bending away from shorter acropodite; both slender and gradually tapering toward tip (Fig. 7A–C). Body colour dark brown, with strong reddish orange paranotal spots (Fig. 16E) (Okinawa-jima Isl., Okinawa Pref.)	***X.rebekae* sp. nov.**
–	Both prefemoral process and acropodite sickle-shaped in mesal view; end of acropodite widening into a lobe (Fig. 9A–C). Body colour grey or dark brown, with strong reddish orange paranotal spots (Fig. 17A, B) (Okinawa-jima Isl. and Kouri-jima Isl., Okinawa Pref.)	***X.variatus* (Pocock, 1895), comb. nov.**

## ﻿Discussion

We present a review of all the species of the genus *Xystodesmus* in East Asia. Based on the smaller body size, the characteristic colour pattern with orange paranotal spots of living specimens, and the gonopod conformation with additional branches as compared to the simple forceps-like gonopods of *Riukiaria*, we describe seven new species from the southern part of Japan, and move two species into the genus *Xystodesmus* (from *Fontaria* and *Rhysodesmus*). The new species from the Ryukyu Archipelago considerably extend the distribution range of the genus to the south. The Korean genus *Koreoaria* with its two species is also synonymised here with *Xystodesmus* on the same morphological basis, and they now represent the first members of the genus on the Asian continent. All the presently known 18 species of *Xystodesmus* are re-evaluated and redescribed. Most of them are illustrated with colour habitus photographs taken of live specimens, to facilitate field identification.

When examining fresh material and literature data, it is clear that, based on gonopodal morphology and molecular phylogenetics, the closest East Asian genus to *Xystodesmus* Cook, 1895 is *Riukiaria* Attems, 1938. Our study, in agreement with Tanabe and Shinohara (1996), showed that in addition to gonopodal characters, live colouration is also important in the separation of the two genera (Korsós 2017). As opposed to the simple, bifurcated, forceps-like gonopod shape observed in most species of *Riukiaria*, *Xystodesmus* species usually have a slightly more complicated forceps-like gonopod with additional branches and appendages. In addition, there is a tendency in body size differences between the two genera as well as other morphological differences, like the presence or absence of metatergal tubercles, and rounded or acute posterolateral corners of paranota. However, variability is high in the two closely related genera, and assignment of already preserved specimens to the appropriate genus based purely on these traits is often remarkably difficult. With a long-standing experience of making field observations of hundreds of live specimens of East Asian xystodesmids, we noted a set of colour characters which seem quite stable and correspond to the generic assignments. The species of the genus *Xystodesmus* usually present uniformly brownish, greyish, or yellowish tergal colouration and lighter paranota, always with bright orange, yellow or pale whitish spots on them, and white legs and antennae whereas *Riukiaria* species are bright orange, yellow, or dark metallic greenish tergal colouration often with dark spots or transverse bands, coloured or dark legs and antennae, and paranotal spots never orange. Recording and carefully describing the live colour patterns of East Asian xystodesmid species whenever possible might add valuable information to their taxonomy.

In the last part of the taxonomic section of their paper, Tanabe and Shinohara (1996) listed seven species which in their opinion possibly belong in *Xystodesmus*. These are *Rhysodesmusspinosissimus* Miyosi, 1952, *Riukiariageniculata* (Takakuwa, 1941), *R.spiralipes* (Takakuwa, 1942), *R.variata* (Pocock, 1895), *Koreoariapallida*, *K.amoena*, and *Pachydesmusbazanensis* Takakuwa, 1942. In the present study, *Riukiariavariata* and the two *Koreoaria* species are now assigned to *Xystodesmus*, but we have little to say about the remaining species. One exception, however, is *R.spiralipes* from Kume-jima Isl. which, according to freshly collected specimens, both in body size, colouration, and gonopod morphology agrees well with the generic characters of *Riukiaria* (and not *Xystodesmus*) as they are considered at the moment. The type material of the Takakuwa’s and the Miyosi’s species being supposedly lost, their exact status could only be settled with the investigation of new, topotypic specimens. *Pachydesmusbazanensis* was transferred to the newly erected genus *Nikkonus* by Chamberlin and Wang (1953), but without type material Tanabe and Shinohara (1996) maintained the combination, albeit *Pachydesmus* Cook, 1895 was excluded as it is a North American xystodesmid genus. Marek et al. (2014) already noticed that because of the body shape and colour described, and the type locality (Masan or Bazan, South Korea), it could be a member of *Xystodesmus*, most probably similar to *X.amoenus* comb. nov., also from the Korean Peninsula.

With respect to the phylogenetic relationship of *Xystodesmus* based on molecular genetic studies, only a few attempts were made. Nakamura and Korsós (2011) presented a maximum likelihood tree with 1063 base pairs of mitochondrial 16S rRNA (part), tRNA-Val, and 12S rRNA (part) genes, and found that *Riukiaria* (15 species) and *Xystodesmus* (9 species) are separated at 85% and 77% confidence levels (1000 bootstrap replicates). Only one species of *Riukiaria* (cf. bifida Takakuwa, 1942), and one of *Xystodesmus* (*fasciatus* sp. nov. in the present paper) were misplaced into the other generic group according to this research (Nakamura and Korsós 2011). In the phylogenetic study of Means et al. (2021b), based on four mitochondrial and two nuclear gene fragments, *Riukiaria* (11 species) and *Xystodesmus* (3 species) were shown to be sister taxa, and also separated from each other and other genera under the tribe Xystodesmini, subfamily Xystodesminae (Means et al. 2021b: fig. 2).

The Ryukyu Archipelago is a subtropical biodiversity hotspot, with many endemic life forms confined to a single or closely connected islands (Otsuka et al. 2000; Government of Japan 2019). The current geographical pattern of terrestrial vertebrates, especially amphibians and reptiles, indicates a strong influence of the formation in geohistory of the archipelago, dividing it into several well-separated island groups (Ota 2000). Millipedes of the family Xystodesmidae are blind soil-dwelling arthropods with limited dispersal ability, thus providing a good potential for historical biogeographical studies. An attempt was already made to consider the geographical distribution of North American xystodesmids (90 species of 20 genera) as an accurate predictor of their evolutionary history (Means and Marek 2017). Their hypothesis was that “geographical proximity may instead be a better predictor of evolutionary relationship than morphology, especially since gonopodal anatomy is extremely divergent and similarities may be masked by evolutionary convergence” (Means and Marek 2017). Their results showed a high degree of morphological convergence in male gonopod shape, and although Euclidean geographical distance was not found to be a better predictor of evolutionary relationship using molecular topology, they concluded that gonopod characters should be viewed critically. When comparing North American to East Asian xystodesmid species, especially to *Xystodesmus* and *Riukiaria*, the similarities in both body shape, colour, and gonopod conformations are striking. One without knowledge of the origins of the samples could easily erroneously assign specimens into the opposite genera, as happened in the past with *Rhysodesmus* and *Pachydesmus*, both now being exclusively New World genera. However, recent molecular genetic studies show, with increasing confidence, that these morphological similarities are clearly the result of homoplasy (Means et al. 2021a, 2021b). A detailed investigation based on a wide taxon sampling in East Asia would certainly be worthwhile to clarify the evolutionary history of the species in the genera *Xystodesmus* and *Riukiaria* with respect to their narrow geographical distribution.

## Supplementary Material

XML Treatment for Xystodesmus

XML Treatment for Xystodesmus fasciatus

XML Treatment for Xystodesmus keramae

XML Treatment for Xystodesmus kumamotoensis

XML Treatment for Xystodesmus kumeensis

XML Treatment for Xystodesmus parvus

XML Treatment for Xystodesmus rebekae

XML Treatment for Xystodesmus sesokoensis

XML Treatment for Xystodesmus variatus

XML Treatment for Xystodesmus pallidus

XML Treatment for Xystodesmus amoenus

XML Treatment for Xystodesmus saltuosus

XML Treatment for Xystodesmus martensii

XML Treatment for Xystodesmus nikkoensis

XML Treatment for Xystodesmus yamamiensis

## ﻿Additional information

### Author ORCIDs

Zoltán Korsós https://orcid.org/0000-0003-1545-5086

### Data availability

All of the data that support the findings of this study are available in the main text.
